# Low-cost food-grade alternatives for serum albumins in FBS-free cell culture media

**DOI:** 10.1038/s41598-025-99603-7

**Published:** 2025-05-01

**Authors:** Lisa Schenzle, Kristina Egger, Bernhard Spangl, Mohamed Hussein, Atefeh Ebrahimian, Harald Kuehnel, Frederico C. Ferreira, Diana M. C. Marques, Beate Berchtold, Nicole Borth, Aleksandra Fuchs, Harald Pichler

**Affiliations:** 1 acib - Austrian Centre of Industrial Biotechnology, Petersgasse 14/V, 8010 Graz, Austria; 2 Institute of Statistics, BOKU University, Vienna, Austria; 3 Department of Applied Life Science, FH-Campus Wien, Bioengineering, Vienna, Austria; 4 Department of Bioengineering and Institute for Bioengineering and Biosciences, Instituto Superior Técnico, Universidade de Lisboa, Av. Rovisco Pais, Lisbon, 1049-001 Portugal; 5 Associate Laboratory i4HB—Institute for Health and Bioeconomy, Instituto Superior Técnico, Universidade de Lisboa, Av. Rovisco Pais, Lisbon, 1049-001 Portugal; 6 Department of Biotechnology, BOKU University, Vienna, Austria; 7 Institute of Molecular Biotechnology, Graz University of Technology, NAWI Graz, BioTechMed Graz, Graz, Austria

**Keywords:** Cultivated meat, Satellite cells, Chinese hamster ovary cells, Serum free media, Albumin alternatives., Biotechnology, Biomimetics, Stem-cell biotechnology

## Abstract

Cultivated meat may be an ethical, environmentally friendly, antibiotic-free meat alternative of the future. As of now, one of the main limiting factors for bringing cultivated meat to the market is the high cost of the cell culture media and their great dependency on serum albumins, production of which is predicted to become a major bottleneck of this industry. Here, using bovine muscle stem cells, we optimized serum free B8/B9 medium. We identified several food grade, low-price medium stabilizers, exhibiting comparable or even superior stabilization of the B8 medium in short- and long-term cultivations, as compared to recombinant human serum albumin. We show transferability of our approach to other satellite cells (porcine, chicken) and CHO cells, though significant cell-line specific differences in response to stabilizers were observed. Thus, we provide an alternative to serum albumin, enabling up to an overall 73% reduction of medium price for certain cell lines.

**Supplementary Information:**

The online version contains supplementary material available at 10.1038/s41598-025-99603-7.

## Introduction

Conventional meat production and especially beef production forms the tip among the most land and emission intensive food products. Since the world population, and consequently meat consumption, is predicted to increase by further 73% until 2050, it will not be possible to obtain enough meat in a conventional way, as already now around 90% of all agricultural land is used for animal breeding^1^. One of the possible alternatives – cultivated meat – can drastically reduce land use and global warming effects.

Cultivated meat has come a long way from its first introduction in 2013 by Mark Post, with his first cultivated meat burger patty at € 250,000.- using expensive and ethically questionable Fetal Bovine Serum (FBS) containing medium – a standard those days, and still broadly used standard medium for biomedical research of myogenic cells^2^. To this day a lot of effort is being put into the development of cultivation medium, as over 95% of the production costs are attributed to it^3,4^. During the last years a lot of progress has been made in this area. Among other – proprietary – solutions^5,6^, three renown groups have published their serum-free, fully defined medium compositions, which allow very high propagation efficiency, comparable to FBS-based medium formulations.

For the first optimized medium, Stout et al. have taken the adapted Essential 8 medium - called B8 medium^6^ – as a basis, and supplemented it with 0.8 g/L human serum albumin (HSA) for stabilization. This new medium, called B9^7^, was shown to perform nearly as well as the 20% FBS-containing growth medium on primary bovine satellite cells (BSCs). Its HSA costs comprised 24.56 USD/L, making up to over 50% of total cost. The other serum-free medium was published by Kolkmann et al.^8^, reaching 97% of the efficiency of the 20% FBS-containing growth medium in short-term experiments. Therefore, Kolkmann used even higher (5 g/L) HSA concentration for an approximate cost of 163 USD/L only for HSA. Yet, Skrivergaard et al. reported very interesting results using a mixture of usual GFs, supplemented with the combination of bovine serum albumin (0.075 g/L) and fetuin (0.6 g/L) – a fetal variant of serum albumin, more abundantly present in FBS than serum albumin^9^.

In addition to nearly the same GFs and signaling molecules that orchestrated the proliferation of stem cells, these media were reinforced by medium stabilization agent albumin, possibly taking on some of the functions of the extracellular matrix (ECM). Such stabilization simultaneously presents a central proliferation potentiating factor, raising effectiveness of the medium, but is also a cost factor, comparable to or even bigger than the GFs themselves. Moreover, the production volume of recombinant albumin required to replace just 1% of the globally consumed meat was estimated to be in the millions of kilograms, surpassing by far the current production volumes of many industrial enzymes^10^.

The most recent paper from Stout et al. has addressed this latter issue, demonstrating a successful HSA substitution by in-house produced seed protein isolates, enriched with plant albumins^11^. Extremely low prices of the oilseed protein meals (less than 0.4 USD/kg), which were used as the starting material, do raise hope that the final prices for protein preparations will be rather low. But it is hard to assess this fact properly, as (i) such isolates are currently commercially unavailable, (ii) the proposed isolation method is quite laborious and could be costly on an industrial scale, and (iii) the isolates must be stored at -80 °C^11^.

Here, we suggest several variants of the improved B8 and B9 media as a serum free, fully defined medium composition, which can be prepared in-house. We present several low-priced, food-grade medium stabilizers and their combinations, which exhibit similar stabilization of the B8 medium as compared to recombinant HSA, allowing for its substitution for some cell lines (most notably porcine, less efficiently - bovine satellite cells). The price for stabilization could thus be lowered by a factor of 370 as compared to B9 medium. Moreover, we show that combination of HSA with methyl cellulose (MC) exhibits a superior stabilization effect for BSCs, as compared to any stabilizer alone, but not for other species’ satellite cells, emphasizing the species-specificity of cell culture medium optimization regarding ECM mimicking. By successful validation of our strategy on Chinese hamster ovary cells, we show broad applicability of these components. In sum, our findings allow for a further cost reduction for the cell culture medium, laying a path for a new direction in the development of a more affordable and sustainable cell culture. It will help to eliminate the bottleneck of a high albumin dependency of the cultivated meat industry in particular, bringing cultivated meat closer to the market, and, potentially, also lowering the production costs of a wide variety of biopharmaceuticals.

## Results

### Single addition of GFs requires much higher concentration for the same effect

GFs are inherently unstable proteins, with some of them having half-lives below 1 h in solution at 37°C^12^. Myokines, which are also known to induce muscle hypertrophy^13,14^, were shown to be rapidly depleted in plasma only 2–4 h after physical activity, and lead to muscle hypertrophy upon repeated physical activity^15^. Considering this, we decided to compare a standard single treatment scheme with a double treatment scheme in the exponential growth phase, which should have simulated a muscle hypertrophic scenario of a repeated “physical activity” with 2 high picks of components and a low basic level in between. Thus, taking B9 medium with 0.8 mg/ml HSA^7^ as a basis, we compared the effect of components’ addition on day 1 with adding them on days 1 and 3 on the proliferation of primary bovine satellite cells (BSCs) (see Supplementary Fig. 1 for characterization of freshly isolated SCs).

**Fig. 1:**
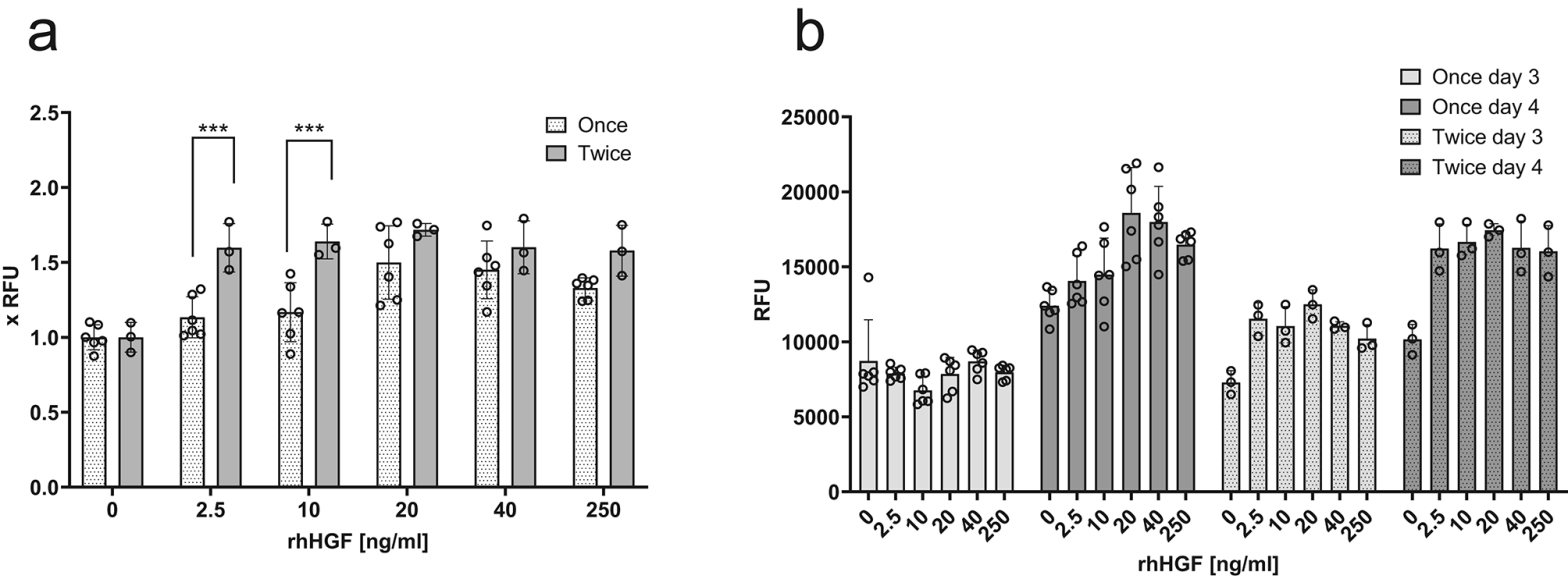
Comparison of single and double addition of rhHGF. BSCs were treated either once on day 1 or twice on days 1 and 3 with indicated concentrations of the rhHGF. Presto Blue assay was performed on days 3 and 4. (**a**) Data of day 4 is presented normalized to vehicle treated control (0), which contained DPBS + 0.8 mg/mL HSA (B9). (**b**) Not normalized data for both days. *n* = 6 for samples treated once, *n* = 3 for samples treated twice; statistical significance was calculated by one-way ANOVA combined with Šídák’s multiple comparisons test, comparing same concentrations in different treatment schemes, and is indicated by asterisks, which are *p* < 0.05 (*), *p* < 0.01 (**), *p* < 0.001 (***).

As expected, single addition did require much higher concentration for the same or less pronounced effect, as we show for recombinant human Hepatocyte Growth Factor (rhHGF). A concentration of 20 ng/mL was necessary for 1.5-fold higher Presto Blue signal (Fig. 1), corresponding to a higher cell density, whereas when applied twice, the threshold of 1.5 fold improvement was consistently achieved at rhHGF concentrations of 2.5 ng/mL (Fig. 1). This emphasizes the significance of adapting the supplementation scheme to conditions that consider instability of GFs. In all subsequent experiments, we applied double addition of GFs.

### Stabilization of cultured medium components by food grade excipients

Albumins are very widely used for non-specific stabilization of biologically active proteins^16–18^. But they are far from being the only known protein stabilizers – salts, sugars, amino acids, fatty acids and hydrogels are also used for this purpose, some of them known under the term “chemical chaperones”^12,19,20^. Different GFs can require various stabilizers at specific concentrations for a significant effect^18,21^. However, sustainable stabilization properties for longer storage are often reached at extremely high, non-physiological concentrations and could be causing serious adverse effects in living cells^22,23^.

Here we investigated the effect of two alternatives to recombinant HSA during exponential growth phase (days 0–4), as well as after the activation of contact inhibition (days 7–10). Methyl cellulose (MC) and racemic alanine (ALA) are known for their non-specific stabilization effects of FGF-2^21^ (one of the components of B8 and B9 media). Both are extremely cheap in comparison to HSA (see Supplementary Table 1), and are food components – MC is an approved food emulsifier also known as E461, and L-ALA is a natural food compound (as opposed to industrially produced racemate, containing equal amounts of left (L)- and right (R)-handed enantiomers). Instead of L-ALA we used racemate to follow-up on the effects published by Benington et al.^21^.

**Fig. 2:**
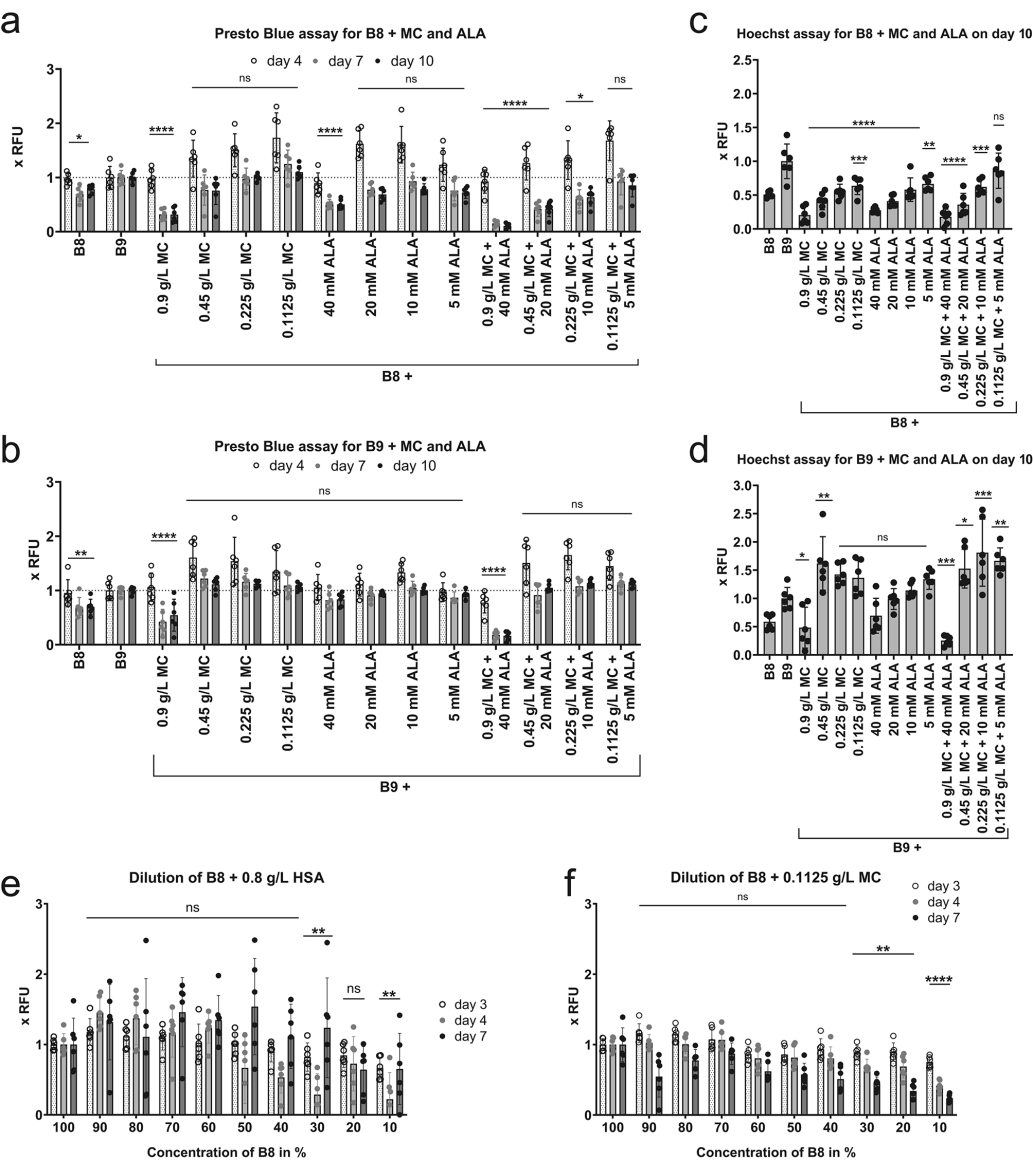
Non-specific stabilization of cultured medium components. 2000 BSCs/cm^2^ were seeded on day 0 in BSC-GM, and changed on day 1 to either B8 or B9 medium, with methyl cellulose (MC) and/or alanine (ALA) added to indicated end-concentrations with every medium exchange. Presto Blue assay was performed on indicated days (**a**,** b**,** e**,** f**), and Hoechst assay – on day 10 (**c** and **d**). Obtained values were normalized to BSCs in B9 (**a**-**d**) or to the values of basic (100%) B8 concentration (**e**-**f**). *n* = 6 and repeated at least twice; statistical significance was calculated by one-way ANOVA combined with Dunnett test for day 7 (Presto Blue **a-b**), for day 4 (Presto Blue **e-f**) or for day 10 (Hoechst), comparing all samples to B9 (**a-d**) or to the values of basic (100%) B8 concentration (**e-f**), and is indicated by asterisks, which are *p* < 0.05 (*), *p* < 0.01 (**), *p* < 0.001 (***), *p* < 0.0001 (****).

In short-term proliferation in supplemented B8 (Fig. 2a, c), both MC and ALA alone could demonstrate significant positive effects as compared to B8, and in combination they were able to stabilize B8 approximately as well as HSA at the lower concentrations used: MC at 0.1125 g/L, ALA at 10 mM and as low as 5 mM (trends supported by Hoechst data, Fig. 2c). Even more interesting was the superior stabilization of B9 medium upon addition of MC or MC + ALA at the lower applied concentrations (Fig. 2b, d). All in all, the most prominent stabilization of B8 – on the level of 0.8 g/L HSA – was achieved by MC (0.1125 g/L) or by the combination MC + ALA (0.1125 g/L + 5 mM respectively). Most prominent stabilization of B9 – at least 1.5x better than B9 - with a triple combination of HSA (0.8 g/L), MC (0.45–0.1125 g/L) and ALA (20 − 5 mM) (Fig. 2d). No morphological changes were detected after cultivation of BSCs for 3 days in B8/B9 supplemented with ALA, MC or combination thereof (Supplementary Fig. 2).

We further investigated if new stabilizers (MC) can potentiate the incorporation of additional growth factors, known to stimulate the proliferation of satellite cells (IL-6^8,25^, Estrogen (17β-Estradiol)^25,26^, Wnt3a and Wnt5b^27^). As shown in Supplementary Fig. 3, in short-term experiments, only B9 + rhHGF + rhPDGF could be further improved by addition of IL-6, but not B8 + MC + rhHGF + rhPDGF. This led us to the decision to focus on stabilization of the media, rather than on identification of further beneficial GFs.

In a further effort to lower the medium costs, we diluted B8 medium containing Insulin, Transferrin, FGF2-G3, TGFβ3, NRG1 as active components, by adding increasing amounts of DMEM/F12, whereas stabilizer concentration was left constant. We show that on day 4 the B8 + MC reduced by 70% and B8 + HSA by 60% did not have any apparent detrimental growth effects. For the later days, the negative effect of dilution is distinct after reduction by more than 30% for MC and more than 40% for HSA (Fig. 2e-f). This effect was generally confirmed by confluency measurements (Supplementary Fig. 4). It is possible that some discrepancies between Presto Blue and confluency assays arise from differences in cell adhesion efficiency to plastic (used in Presto Blue) versus glass (used in confluency) surfaces, as well as from confluency imaging performed at the wells’ centre, analysing approx. 30% of the well, in order to avoid lens effect at the edges, distorting the proportions. Imaging analysis additionally confirmed, that 30% reduction does not affect the morphology of the cells (Supplementary Fig. 4). The confluency assessment further shows that cell density on days 3 and 4 remains significantly below contact inhibition levels, while on day 7, some conditions may achieve over 70% confluency.

### Stabilizers mechanism of action

First, we have investigated whether the degradation of GFs is influenced by the addition of stabilizers MC and L-ALA at such low concentrations.

**Fig. 3:**
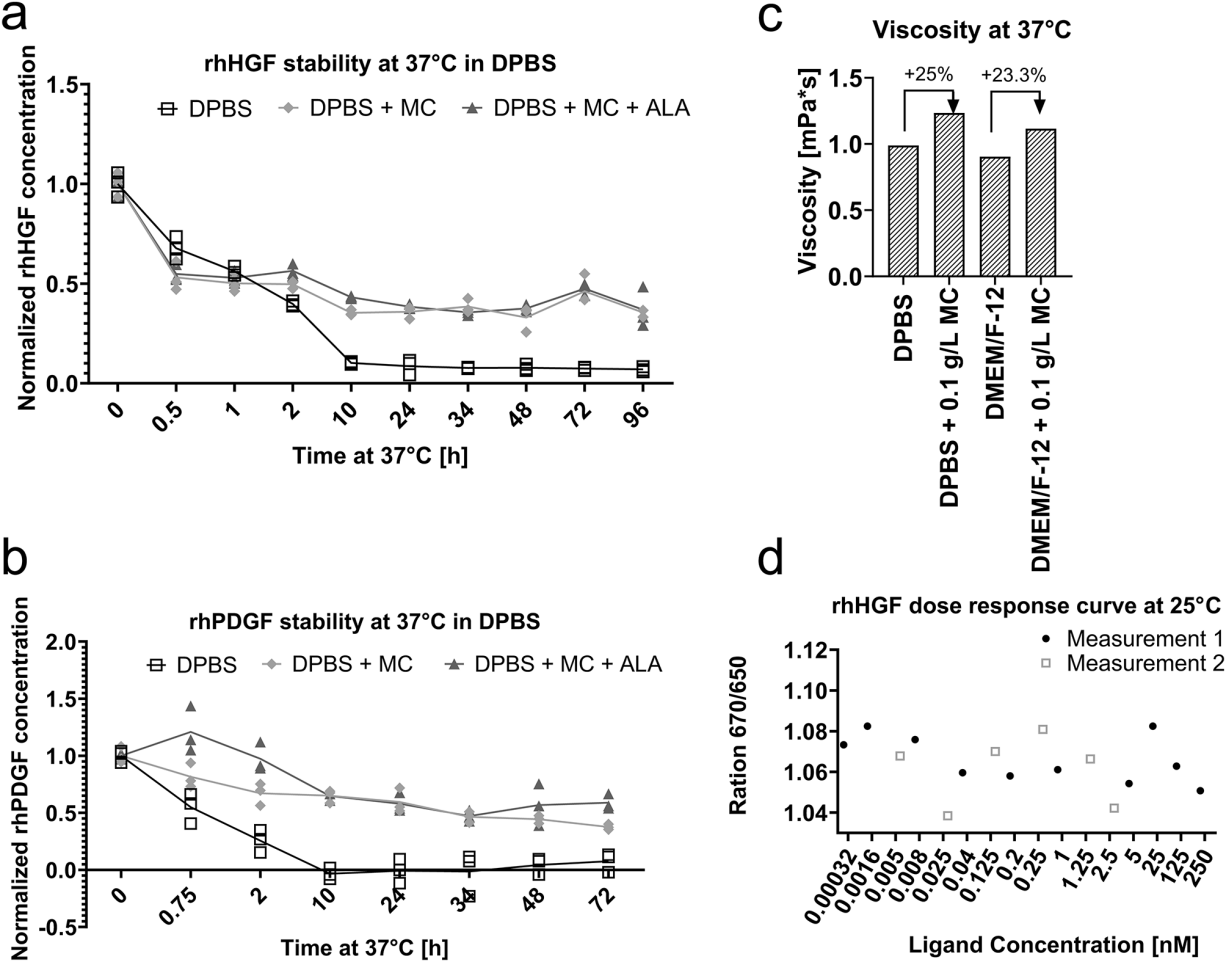
GFs in DPBS were stabilized either with 0.1 g/L MC or 0.1 g/L MC and 5 mM ALA. Stability of rhHGF and rhPDGF at 37 °C in DPBS was measured by ELISA following manufacturer’s manuals (**a** and **b**). GFs in DPBS were stabilized either with 0.1 g/L MC or 5 mM ALA. DPBS without stabilizers was used as control. All samples were aliquoted on ice, incubated for the indicated time at 37 °C, then frozen (including time point 0), and ELISA was performed after freezing the last sample. (**c**) Viscosity of the buffers and media at 37 °C with/without 0.1 g/L MC. (**d**) Change in rhHGF conformation through direct interaction with MC as ligand was not detected.

rhHGF half-life of 1 h in DPBS at 37 °C was slightly higher than that of rhPDGF with 45 min (Fig. 3a and b), and the degradation of both rhHGF and rhPDGF was partly circumvented by the addition of MC at 0.1 g/L concentration.

High media viscosity is known to contribute to a higher cell survival by acting as an inert adhesion-promoting matrix, possibly through the induction of a higher actin density and remodelling of cytoskeleton^28^. But on the other hand, enhanced viscosity could be disadvantageous for quickly proliferating cells, e.g. as shown in Fig. 2a-b. We further compared the viscosity of our solutions. The viscosity of 0.1 g/L MC solution was approximately 25% higher than that of background medium (Fig. 3c) and could possibly play a role in higher proliferation by promoting cell adhesion.

Another aspect of stabilizers’ action is reducing the protein’s free energy through interactions such as ionic, electrostatic, and hydrophobic interactions^21,29^. Specific binding of ligands tends to stabilize a variety of proteins – from enzymes to GFs^30–33^, thus we investigated whether the stabilizing effect of methyl cellulose on GFs is due to such high energy interactions. We investigated the direct interaction between rhHGF and MC as it has demonstrated a more pronounced effect on rhHGF stability. We used NanoTemper Thermophoresis assay to measure the change in GF conformation upon binding to MC. Yet, as shown in Fig. 3d, there seem to be no apparent trend in conformational change of rhHGF in the wide range of MC concentrations (0.00032 nM to 250 nM).

### Screening for further alternative stabilizers

Recently, concerns have been raised about the presence of MC in food items due to its synthetic nature^34^, as it is manufactured by heating cellulose with caustic soda and subsequently treating it with methyl chloride^35^. We thus explored other possible stabilizers, which would not raise questions about their inclusion into the final food product – e.g. food-grade polysaccharides and sugar alcohols, with certain structural similarity to methyl cellulose. We also took into account the option of using substances that are metabolically inert (in contrast to ALA).

We identified 3 polysaccharides/sugar alcohols with a similar effect on proliferation rates, as compared to B9 or B8 + MC (Supplementary Fig. 5) – sorbitol, starch from corn/rice, and locust bean gum. The latter was not included into further experiments due to extremely high viscosity even at low concentrations and tendency to produce aggregates, which led to inconsistent handling and ill reproducibility.

We employed design of experiments (DoE) methodology to uncover potential synergies among combinations of stabilizers and to fine-tune their concentrations. Space-filling design^36^ with 6 replicates per stabilizer combination was applied in a short-term proliferation experiment with 6 best stabilizers in concentration ranges listed in Supplementary Table 2 (see experimental setup in Supplementary Table 3). Combinations of 3 components at maximum concentrations were excluded from the design to avoid possible adverse effects of higher viscosity. Proliferation of BSCs was quantified using Presto Blue assay, normalized to B9, and analysed using R^37^ with fitting of linear and linear mixed effects models of second order by stepwise forward regression. Additionally, raw- and background corrected data was analysed in the same way, yielding very similar outputs.

Cell density was chosen as an aim of optimization. Influence of stabilizers on proliferation and their reciprocal effects were analysed on single days (days 4, 6 and 8) separately, as well as combined (Supplementary Table 4). Among single components, MC was the only component which yielded significant response on the two of three single days (days 4 and 6), whereas starch from corn (STC^2, the quadratic term of STC – see Supplementary Table 4) was significantly advantageous only on day 1. Also, only MC and STC^2 effects reached significance in the analysis of all three days.

**Fig. 4:**
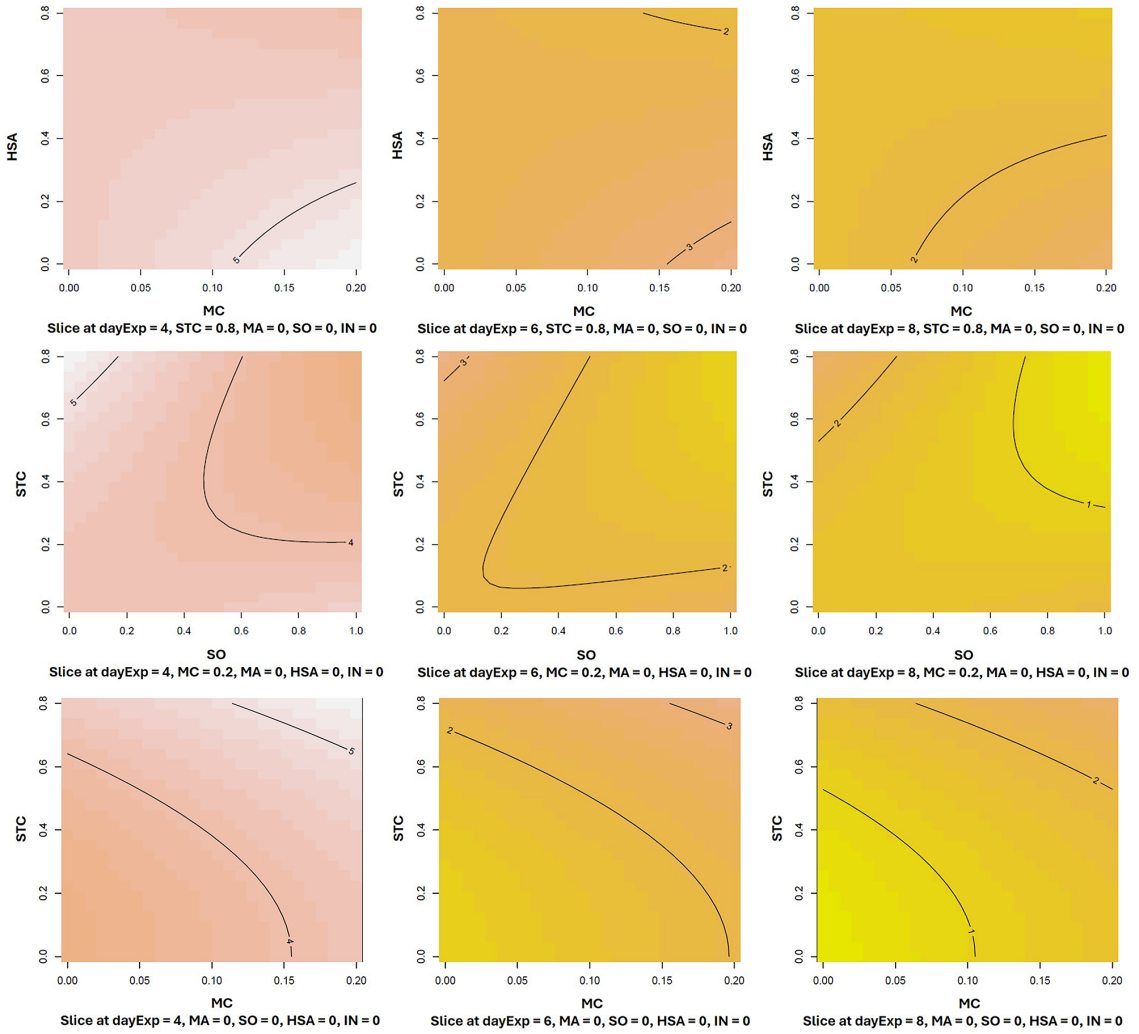
Response surface contour plot predicting the cell density as a function of stabilizer combinations for days 4–8. Optimization of stabilizers concentrations was performed in a short-term proliferation experiment, where starch from corn (STC), recombinant human serum albumin (HSA), methyl cellulose (MC), sorbitol (SO), mannitol (MA) and inulin (IN) were applied in combinations at concentrations listed in Supplementary Tables 2 and depicted on the axes in g/L. Experimental setup is depicted in Supplementary Table 3. The statistics software R^37^ was used for the analysis of the normalized to B9 values. The experiment was repeated twice, both runs used for modelling.

We have also analyzed cell density as a function of possible interplay of multiple components, and most significant effects of the model, predicting the proliferative effects throughout days 4 to 8, are presented on Fig. 4. In this model, the most advantageous conditions were in higher concentrations of single components, though several components (STC, MC, SO) have demonstrated additive positive effects. From the combinations of 6 tested components, only MC and SO have shown somewhat synergistic effect on day 8 (MC: SO), but without reaching significance – *p* = 0.19 (Supplementary Table 4). Interestingly, combination MC: HSA, which seemed more advantageous than MC alone in short-term (Fig. 2a-b), was shown here rather adversary, with the same trend of favourable effects in cases of either HSA of MC alone.

**Fig. 5:**
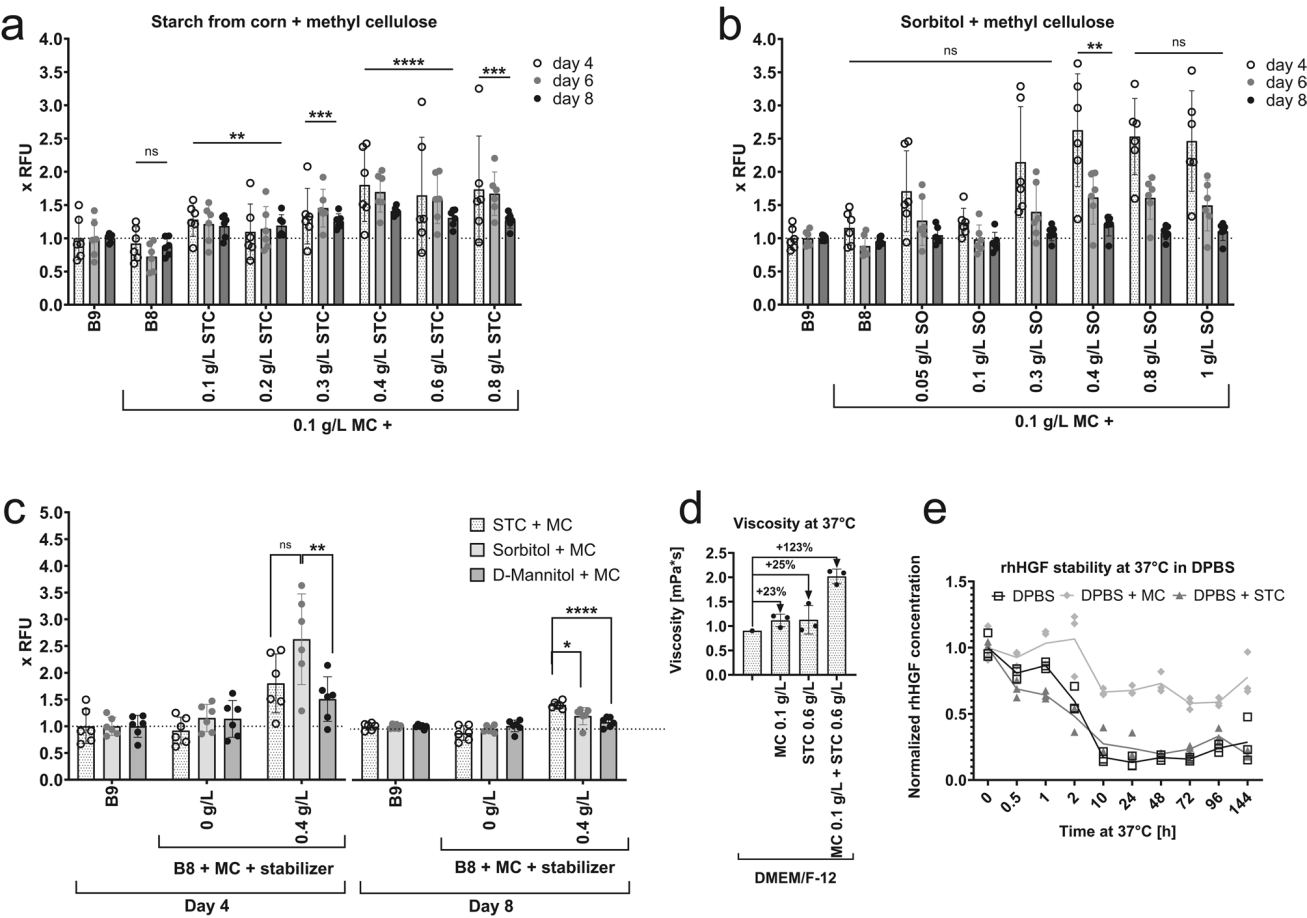
Further non-specific stabilizers with MC in B8. (**a**-**c**) 2000 BSCs/cm^2^ were seeded on day 0 in BSC-GM, and changed on day 1 to the designated medium. B8 with 0.1 g/L methyl cellulose (MC) and B9 medium were used as controls. Stabilizers were added to indicated end-concentrations with every medium exchange, MC always 0.1 g/L. Presto Blue assay was performed on indicated days. Obtained values were normalized to BSCs in B9. *n* = 6 and repeated at least twice; statistical significance was calculated by one-way ANOVA combined with Dunnett test for day 8 (a-b) or day 4 and day 8 (c), comparing all samples to 100% B9 (a-b), and is indicated by asterisks, which are *p* < 0.05 (*), *p* < 0.01 (**), *p* < 0.001 (***), *p* < 0.0001 (****). (**d**) Viscosity of the DMEM/F12 media with/without 0.1 g/L MC and/or 0.6 g/L STC, each measurement except of pure DMEM/F12 was repeated thrice. (**e**) Stability of rhHGF at 37 °C in DPBS was measured by ELISA following manufacturer’s manuals. rhHGF in DPBS was stabilized either with 0.1 g/L MC or 0.4 g/L STC. DPBS without stabilizers was used as control. All samples were aliquoted on ice, incubated for the indicated time at 37 °C, then frozen (including time point 0), and ELISA was performed after freezing the last sample.

To verify the DoE experiments, we have compared the combinations predicted to be advantageous in a standard short-term proliferation experiment (Fig. 5a-c and Supplementary Fig. 6). Interestingly, the combination of MC with STC in B8 has shown most stable proliferation induction, significantly exceeding that of B9 or B8 + MC both in exponential phase (day 4), as well as upon activation of contact inhibition (day 8, Fig. 5a). Sorbitol + MC (Fig. 5b-c) and inulin + MC (Supplementary Fig. 6) have shown comparable effects to STC + MC in exponential phase (day 4).

We have further measured the viscosity of the best stabilizer mix 0.1 g/L MC + 0.6 g/L STC, which appeared approx. 100% higher than MC and STC on their own (Fig. 5d), demonstrating more than additive increase. This much higher viscosity could play a more significant role in higher proliferation rate of BSCs through physical properties rather than by stabilizing GFs. This conclusion was further supported by the fact that STC does not demonstrate a stabilizing effect on rhHGF, in contrast to MC (Fig. 5e).

### Long-term proliferation of BSCs is improved in stabilized medium

One of the most important criteria to assess the efficiency of the new media are long-term proliferation experiments, where cell count is monitored upon every passage and myogenic potential of the cells grown and differentiated in standard or stabilized medium is assessed by calculating fusion index in differentiated cells after each passage. To validate our short-term results, we have performed such experiments for bovine satellite cells in optimized B8 and B9 media.

**Fig. 6:**
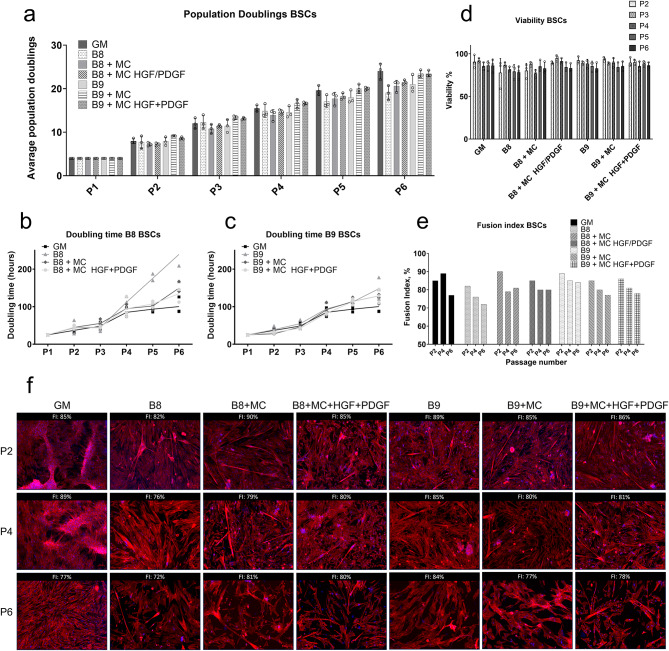
Long-term proliferation experiments with BSCs in stabilized B8 and B9. Cells were cultured in 6-well plates in corresponding media, with 0.1 g/L MC and 2.5 ng/ml GFs rhHGF and PDGF added where indicated immediately upon splitting, whereas HSA was added 24 h after the splitting. Average population doublings P1 = 4 was assessed based on cells count after isolation. Cells were stained with trypan blue and counted upon passaging using Invitrogen Cell Countess, and the number of viable cells (**d**) was used to calculate population doublings and doubling time (**a-c**). Upon each passage, part of the cells was seeded for differentiation and IF staining (**f**) and fusion indices were calculated based on the ratio of cells with at least two nuclei to all nuclei. A range of 250–1700 nuclei were counted for each sample. The DAPI count was performed by the Zeiss Zen tool for cell counting (**e**). Immunofluorescence staining was performed for nuclei (DAPI, blue), desmin (Supplementary Fig. 7) and actin (phalloidin, red). Cells were grown to confluency and differentiated for 10 days in a serum-free differentiation medium (see Materials and Methods), with addition of stabilizers, if they were present in propagation medium. Scale bar = 100 μm. Microscope Zeis Axio Imager. Lens: EC Plan-Neofluar 10x; Exposure DAPI: 20 ms, Phalloidin: 300 ms. GM = growth medium, FI = Fusion Index.

We conclude that additional stabilization with MC was successful in reaching higher population doublings in stabilized B8 in later passages and stabilized B9 media from the beginning, as compared to B8 and B9 without MC (Fig. 6). BSCs’ population doublings in B9 + 0.1 g/L MC, as well as doubling time were comparable with GM. Additional supplementation with rhHGF and rhPDGF did show a positive, but insignificant trend on proliferation in case of B8, but not B9. Best stabilizer of differentiation medium was HSA, reaching the highest fusion indices, whereas addition of MC seemed to have a rather negative effect on differentiation efficiency of cells in HSA supplemented medium, resulting in shorter myofibers and lower fusion index. This effect is reversed in HSA-free medium, where both parameters improved upon MC supplementation (Fig. 6e-f). These results could be explained by the stabilization/retention effect MC and HSA have on different GFs in the differentiation and probably also propagation media. This would need to be further explored.

We have investigated the potential of the other two strategies to further lower BSCs cell culture media price, identified in the short-term experiments: the combination of MC and STC and dilution of the growth factors to 70% from the original concentration. Addition of 0.4 g/L STC to B8 + MC could further slightly increase final population doublings, quite up to the level of B9 + MC, by improving viability of the cells (Supplementary Fig. 8e and b). In B8 + MC, addition of STC also promoted fusion of the myoblasts upon differentiation with no effect on fiber length or thickness, as compared to B8 + MC (Supplementary Fig. 8g).

In the long-term proliferation, addition of stabilizers could as well almost completely compensate the dilution of the B8 and B9 media to 70% from the original concentration. Also, propagation in diluted stabilized media led to thicker and longer muscle fibers upon differentiation in identical differentiation medium (e.g. B8 + MC compared to 70% B8 + MC in Supplementary Fig. 8g), again supporting our assumption that GFs from propagation medium are partly preserved in culture throughout medium exchanges, thus decreasing the efficiency of muscle fiber maturation.

### Stabilized medium is applicable for other relevant cell lines

Protein content of the ECM has largely formed in early chordates prior to the emergence of vertebrates^38^, leading to the assumption that the overall molecular content is somewhat similar to our days. We thus hypothesized that our stabilizer mixtures could probably have the same effect on proliferation of muscle precursor cells of other species. To investigate that, we have conducted proliferation experiments with porcine (PSCs), chicken (CSCs) and fish satellite cells.

Interestingly, in contrast to BSCs, the most successful long-term proliferation medium for PSCs and CSCs appeared to be HSA free B8 + MC (Supplementary Fig. 9 and Supplementary Fig. 10). Additional supplementation of B8 with rhHGF + rhPDGF showed a positive trend on proliferation and cell viability for PSCs, but not for CSCs. As in case of BSCs, HSA – supplemented differentiation medium was leading to a higher fusion index and longer muscle fibres of differentiated PSCs, indicating diverging media requirements for proliferation and differentiation phases of the same cell line.

Despite recent developments in serum-free culture media for mammalian cell lines, only a few studies on serum-free media exist for fish cells. The majority of studies was performed in cell lines which are not relevant for cultivated fish^39,40^. Therefore, we explored the potential of stabilized B8 culture media in field-relevant cell lines such as Atlantic mackerel (*Scomber scombrus*) skeletal muscle cell line (MACK1)^41^ and European bass (*Dicentrarchus labrax*) Embryonic-like Cell Line (DLEC)^42^. As shown in Supplementary Fig. 11, B8 medium has a higher potential to be used for MACK1 cell line, than for DLEC cell line, though the fact that we could not reach a complete substitution of FBS leads to conclusion that B8 lacks GFs, crucial for fish cell lines. For MACK1 cells, supplementation of the medium with 0.1 g/L MC and/or 0.8 g/L HSA had rather a negative effect on proliferation, though no morphological changes were detected.

### Stabilized medium is applicable for industrially relevant cell lines

The Chinese hamster ovary (CHO) cell line^43^ is widely used in research and is a workhorse of the biotechnological industry, famous for the production of a spectrum of recombinant products, e.g. GFs^44–46^. We have therefore speculated that the ability of MC/HSA to stabilize proteins at low concentrations could also be applied in CHO cell culture to either promote cell proliferation or to stabilize the products.

Serum free CD-CHO growth medium was supplemented with either 0.1 g/L MC or 0.1 g/L MC with 0.8 g/L HSA. An Epo-Fc producing CHO-DUXB11 line was adapted to these new media compositions for 21 days by passaging in supplemented growth medium every 3–4 days. After adaptation, cells were banked, and a small-scale batch culture was performed in three replicates to assess whether the additives had an impact on cell growth and viability. In addition, supernatant samples were measured using Octet RED96e (ForteBio) every 24 h to assess the Epo-Fc titers.

**Fig. 7:**
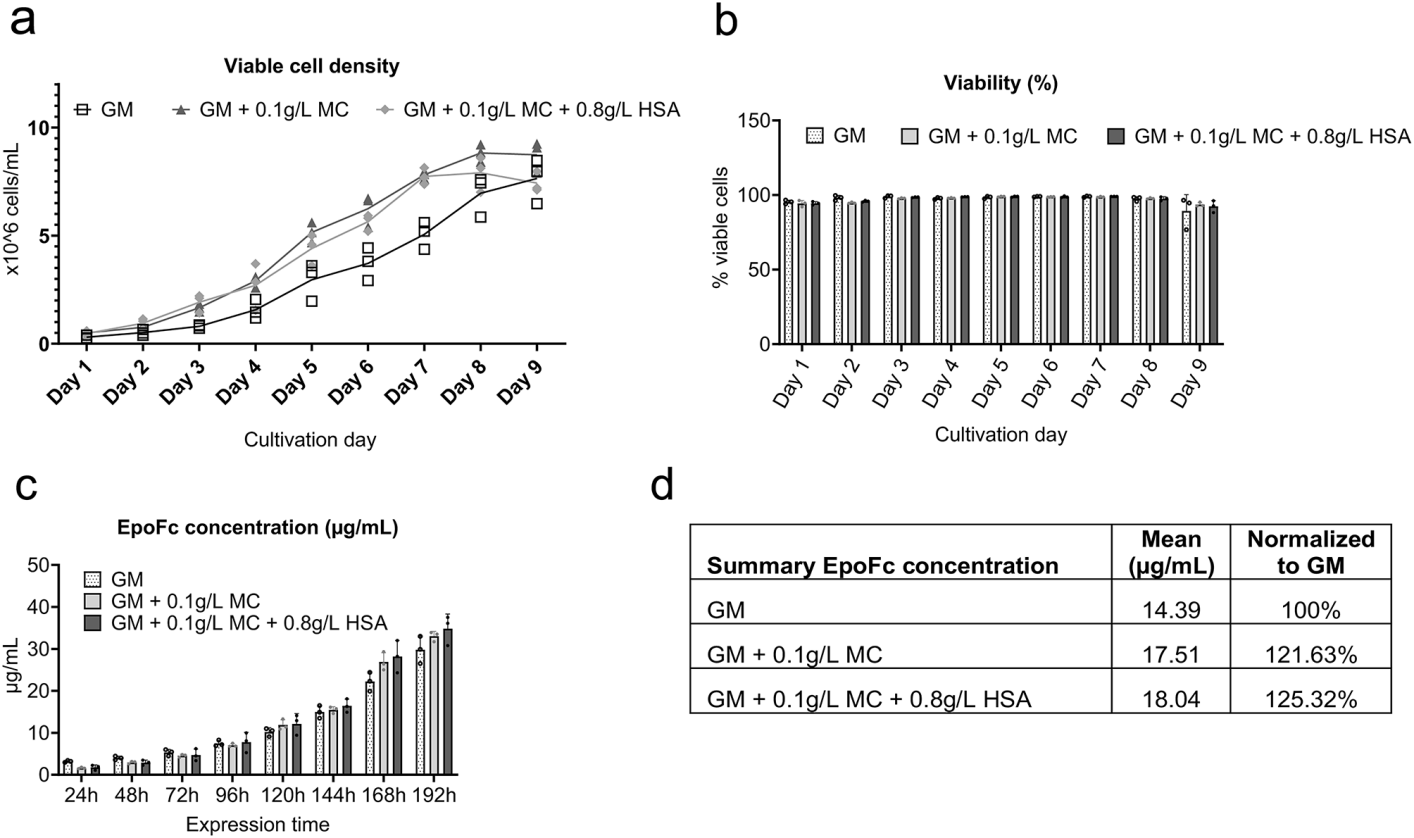
Effect of MC and HSA on expression of EpoFc in CHO-DUXB11 cell line. (**a**) Viable cell density (VCD) and viability (**b**) of CHO-DUXB11 expressing human erythropoietin fusion protein (EpoFc) in small-scale batches with the addition of stabilizers. (**c-d**) EpoFc titer concentrations from sampled supernatant of the batch cultures. Cultivations were performed in biological triplicates.

From day 5 on (120 h) the gap in titers between the MC containing samples and GM increased, which can be explained by MC having a positive effect on the cell viability (Fig. 7a-d). At the tested concentrations, none of the additives affected the binding of the EpoFc to the Octet sensors (see Supplementary Fig. 12), which indicates that such additives would have minimal effect, if any, on the downstream processing.

We have further applied the same strategy to Vero cells – one more industrially relevant cell line widely used as a substrate for human vaccine manufacturing^47^. As shown in Supplementary Fig. 13, a slightly negative effect was identified upon addition of higher concentrations of MC in B8 and commercial medium VPSFM. But, interestingly enough, B8 could successfully substitute the commercial VPSFM medium, which is substantially more expensive, and the dilution strategy has demonstrated that both 80% and 70% of VPSFM promoted significantly higher proliferation rates than 100% VPSFM.

## Discussion

Development of the serum free medium for expansion of primary satellite cells has come to a new level in the last two years, as several medium compositions were presented demonstrating efficiency comparable to FBS containing medium compositions. This was facilitated by observing the importance of using not only the right combination of GFs, but also after introduction of medium stabilization. Stout et al.^48^ and Kolkmann et al.^8^ use well known and very widely applied human or bovine serum albumin for medium stabilization, Skrivergaard et al.^9^ also, but in combination with fetuin. In these examples, about 61–81% of the media costs are attributed to stabilizers, posing a new challenge for cultivated meat industry, as they are not only pricy, but also applied in high concentrations (see Supplementary Table 5 for media price comparison). Recent evaluations of recombinant protein costs and volumes necessary for cost-competitive cultivated meat production suggest that 96.6% of production volume is expected to be attributable to albumins, and a little less than 4% will comprise transferrin, insulin, growth factors and other proteins^10^. Based on these assumptions, to produce cultivated meat amounting to 1% of the global meat market would require millions of kilograms of albumin, which by far exceeds the current production volumes of many recombinant industrial enzymes, and of the serum derived albumins as well. In a recent paper Stout et al. presented the possibility to substitute HSA with seed protein isolates, which are not yet commercially available, but can be produced in-house^49^. The costs of this solution are yet to be evaluated, including the requirement of storage at -80 °C for the isolates to stay biologically active.

But although albumins are very widely used for non-specific stabilization of biologically active proteins^16–18^, they are far from being the only known protein stabilizers – salts, sugars, amino acids and hydrogels are also used for this purpose, some of them known under the term “chemical chaperones”, and are usually utilized for refolding of recombinant proteins from inclusion bodies, or for storing proteins over longer time periods^12,19,50^.

We have therefore chosen several stabilizers, which are readily available in big quantities, are low-priced in production and storage, and possess extremely long shelf-lives. We have narrowed down to several components based on the published literature, focusing on stabilization of the GFs present in B8/B9 media, and complemented them with potential stabilizers based on structural similarity to stabilizers known to effectively prolong half-life of crucial GFs (Supplementary Table 1).

In this study we have shown that numerous known and new protein stabilizers and their combinations can be used in cell culture – methyl cellulose (MC), alanine (ALA), starch from corn (STC), etc^21,51^ – instead of or together with HSA for a comparable stabilization effect, and that the final combination of stabilizers is cell line specific. Our results are in line with the FGF-2 stabilization study from Benington et al., who demonstrated that a combination of two stabilizers – MC + ALA or MC + HSA – performs for FGF2 much better than any component alone^21^.

High concentrations of MC are traditionally used in various cell culture methods, such as preservation of cell function in suspension^52^, cell aggregation for 3D spheroid formation^53^, or colony forming cell assay^54^, where concentrations from 3 to 0.2% methyl cellulose are used. It is also widely used in food industry (registered as E461 in the EU) as thickener, emulsifier and for its binding and gelling properties – e.g. in plant-based meat alternatives^34^, which was recently criticized^34^ due to its’ synthetic nature^35^. STC, on the other hand, is a naturally occurring edible polymer with the same various applications in the food industry as thickener, stabilizer and binder, which is also used as a hydrogel-polymer^55^ and a drug delivery system^56^. We could show that both substances were exerting a significantly positive effect on BSCs proliferation. MC was also able to improve the stability of one of the crucial GFs – rhHGF, although there seemed to be no specific binding of the GFs and stabilizer present, as it is known for other well described stabilizers of GFs, e.g. heparin^12^. We thus conclude that the mechanisms of action of MC, and possibly of STC, might be based on the weaker interactions of crowder’s effect, leading to a longer half-life of some proteins. Similar effect is known for the favorite molecule of the nanomedicine and another broadly used chemically manufactured food and cosmetics additive – polyethylene glycol (PEG)^57^. In this regard, high concentrations of such largely “inert” macromolecules could be mimicking the ECM environment, i.e. limited space containing only restricted amounts of free water, filled mostly with long polysaccharide chains of glycosaminoglycans and with fibrous proteins^58^. Additionally, it could be a result of the improvement of cell adhesion through increase in media viscosity.

On a molecular level, it is known that MC, as well as other polymeric stabilizers such as PEG, sequesters hydrophilic proteins, preventing their aggregation and subsequent decay, whereas HSA stabilizes through ionic, electrostatic and hydrophobic interactions^16,18^. We also applied ALA in the present study. Amino acids with no net charge, e.g. alanine and glycine, are often used as cryoprotectants of proteins, providing stability through weak electrostatic interactions^59^.

**Fig. 8:**
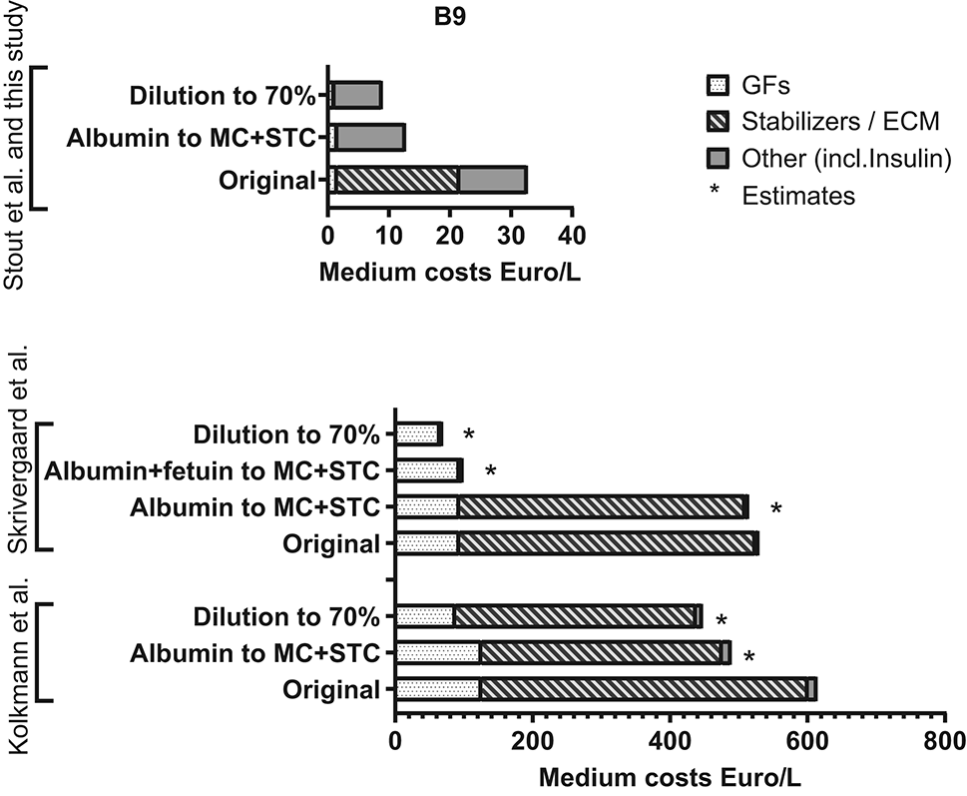
The potential of alternative protein stabilizers and of dilution strategy to reduce medium costs. Medium cost calculations are based on bulk prices summed up in Supplementary Table 5. GFs = growth factors; ECM = extracellular matrix; MC = methyl cellulose; STC = starch from corn.

The main goal of this work was to establish strategies for lowering the price for cultivation medium. Stabilizing the media with newly identified ECM-mimicking components, such as MC and STC, allowed us to substitute HSA for certain cell lines: for PSCs with B8 + MC (Supplementary Fig. 9) and – slightly less efficiently - BSCs with B8 + MC + STC (Supplementary Fig. 8e). New stabilizers also allow reduction of the initial concentration of B8/B9 components to 70% from the original without losses in BSCs cell density, leading to a 73% combined price reduction (Fig. 8 and Supplementary Table 5) and a more efficient muscle fiber maturation (Supplementary Fig. 8g). Additional research is needed to validate that approx. 20% higher viscosity, caused by the addition of single stabilizers, does not lead to a substantial raise in energy consumption in stirred bioreactors, and does not affect heat distribution, hampering cooling. Also, stabilizers’ influence on different growth factors is quite specific, complicating transfer of the media optimization results to other cell lines^18,21^. This was also confirmed in our study, as efficiency of stabilizer combinations diverged for BSCs, PSCs and CSCs, and none of the final tested combinations was successful in improving fish cell lines’ proliferation, or proliferation of Vero cell line.

Strikingly, for both porcine and bovine satellite cells, B8 was not the best performing, but still a possible long-term cultivation medium, whereas Stout et al. have reported, that it was impossible to cultivate their BSCs for longer than two passages^7^. It might be due to the sourcing discrepancy between the cells – we were unable to source samples from 1 to 2 months old bulls, and were constrained to approx. 1.5 years old animals instead. This might have led to a higher flexibility regarding media requirements of the more mature satellite cells, but can have other disadvantages – for example, higher doubling times in the later passages, than those reported for the satellite cells from 1 to 2 month bulls. For the P5 cells doubling times of approx. 75 h if sourced from an older animal was observed, as compare to around 50 h, if sourced from a younger animal^7^. This is in line with other reports on the significantly higher doubling time of the satellite cells’ isolated from older animals – e.g. mice^60^, pigs^61^ and humans^62^.

In conclusion, this and further research will mitigate or eliminate the bottleneck of a high albumin dependency of the cultivated meat industry and will allow a faster upscaling of the technology by making massive investments into additional infrastructure for recombinant albumin production obsolete. Our findings can also be transferred to medical research to support the efforts of 3R concept of minimize animal use (Replacement, Reduction, and Refinement)^63^ by producing a more cost-effective media. Another line of possible application is in pharmaceutical industry in production of various therapeutical proteins, as shown in this study on the example of EpoFc production in CHO-DUXB11 cell line. This could lead to a more affordable medicine.

## Materials and methods

### Materials

rhHGF ELISA kit #BMS2069INST and rhPDGF ELISA kit #BMS2071 were obtained from Thermo Fisher. Monolith His-Tag Labeling Kit RED-tris-NTA 2nd Generation kit (Cat#MO-L018). B8 (HiDef-B8 500X) was purchased from Defined Bioscience (#LSS-201). For sourcing stabilizers, GFs, hormones and cytokines see Supplementary Table 1. Epo-Fc producing CHO-DUXB11 cell line was produced in the laboratory of the co-author Nicole Borth^64^. MACK1 cell line was purchased (kerafast #ETU008-FP). DLEC cell line was purchased (kerafast #ETS001).

### Isolation of satellite cells

Bovine Satellite Cells (BSCs) were isolated from 19 months old, castrated Simmental Oxes (*Bos taurus*). *M. semitendinosus* muscle tissue sample was provided by Marcher Fleischwerke GmbH. Porcine Satellite Cells (PSCs) isolated from 6 month old males *Sus scrofa domestica*,* M. semitendinosus* muscle tissue sample provided by Marcher Fleischwerke GmbH. CSCs = Chicken Satellite Cells isolated from a *Gastrocnemius* of a 5 month old male rooster provided by Thomas Raberl. Cells were isolated from sacrificed animals using a standard protocol described by Stout et al.^65^

A sample the size of ~ 0.5 g of skeletal muscle tissue was extracted. The muscle probe was transported in transport medium on ice and immediately further processed, where it was cut up into small pieces and digested in 0.2% collagenase II (Worthington Biochemical #LS004176, Lakewood, NJ, USA; 275 U/mg) for 45–60 min until the paste was homogenized. Digestion reaction was brought to a halt with BSC growth medium (P-GM). The cells were filtered twice, counted using an Invitrogen Countess Automated Cell Counter and plated at a density of 100,000 cells/cm^2^ onto uncoated tissue-culture flasks. Throughout the incubation over 24 h at 37 °C with 5% CO_2_ adherent cells attached at the surface of the tissue-culture flask and SCs stayed in suspension and were transferred to coated flasks at a density of 2000 cells/cm^2^ with 1.5 µg/cm^2^ recombinant human Vitronectin (FisherScientific #15134499). SCs were left untouched for three to four days before P-GM was changed every two days until a maximum of 70% confluence was reached and the cells were frozen in FBS with 10% dimethyl sulfoxide (DMSO, Sigma #D2650) or passaged for screening or differentiation by using 0.25% trypsin-EDTA (ThermoFisher #25200056).

### Confirmation of SCs identity and differentiation

After isolation, undifferentiated SCs were identified by staining for Paired-box 7 (Pax7) marker. SCs were cultured in BSC growth medium (BSC-GM) on a cover glass until they reached 70% confluence, fixed with 4% paraformaldehyde (FisherScientific #AAJ61899AK) for 30 min, washed in DPBS, permeabilized for 15 min with 0.5% Triton-X (Sigma #T8787) in DPBS, blocked for 45 min with 5% goat serum (ThermoFisher #16210064) in DPBS with 0.05% sodium azide (Sigma #S2002), and washed with DPBS with 0.1% Tween-20 (Sigma #P1379). Primary Pax7 antibodies (ThermoFisher #PA5-68506) were added at a dilution of 1:500 in blocking solution (Aligent Antibody Diluent #S202230-2) containing 1:100 Phalloidin 594 (ThermoFisher #A12381) to cells and incubated at 4 °C overnight. Further, cells were washed with DPBS + Tween-20 and incubated with secondary antibodies for Pax7 (ThermoFisher #A-11008, 1:500) for 1 h at room temperature, washed with DPBS + Tween-20 and mounted with Fluoroshield mounting medium with DAPI (Abcam #ab104139). Visualization and imaging were performed by the Core Facility Imaging at Medical University Graz to validate the satellite cell purity of the isolated cell population.

Isolated SCs were differentiated for ten days. After growing cells to confluency in BSC-GM, the medium was changed to Differentiation Medium (DM) and then 50% of the medium was regularly changed (every two to three days) until SCs reached differentiated state and were fixed and prepared as previously described. Phalloidin 594 was diluted 1:100 in blocking solution (Aligent Antibody Diluent #S202230-2) and incubated at 4 °C overnight. Fluoroshield mounting medium with DAPI was mounted next day. For determination of the fusion index Phalloidin stained cells were used, and the number of DAPI stained nuclei assessed using ZEISS ZEN cell counting module. After that cells containing more than 2 nuclei per cell were manually counted^66^.

### Media composition

Skeletal muscle tissue sample was transferred into transport medium consisting of DMEM + Glutamax (ThermoFisher #10566016) and 1% Antibiotic-Antimycotic (ThermoFisher #15240062) after extraction. For Isolation of BSCs, Primocin growth medium (P-GM) was used containing DMEM + Glutamax (ThermoFisher #10566016), 1% Primocin (Invivogen #ant-pm-1), 20% fetal bovine serum (FBS; ThermoFisher #26140079) and 1 ng/mL human FGF basic/FGF2/bFGF (R&D Systems #233-FB-025/CF). After passage one, P-GM was changed to growth medium (GM) where Primocin is replaced by 1% Antibiotic-Antimycotic (ThermoFisher #15240062). Serum free differentiation medium was prepared according to Messmer et al.^66^.

For short-term and long-term growth analysis, cells were plated in 96-well (Greiner #655180) or 6-well tissue culture plates (Greiner #657160) containing BSC-GM with 1.5 µg/cm^2^ recombinant human Vitronectin (FisherScientific #15134499). After incubation for 24 h, cells were washed with DPBS (ThermoFisher #14190250) and BSC-GM was changed to screening medium - B8 medium containing DMEM/F12 (ThermoFisher #11320033), HiDef-B8 medium aliquots (Defined Bioscience #LSS-201) and 1% Antibiotic-Antimycotic, supplemented or not supplemented with 0.8 g/L HSA. According to the screening method, defined concentrations of medium components and stabilizers (Suppl. Table 1) were added. All added GFs were reconstituted and diluted in DPBS containing 0.8 g/L HSA (Sigma #A9731-1G), highly concentrated aliquots were stored at -80 °C.

### Screening of BSCs/PSCs/CSCs

BSCs/PSCs/CSCs were thawed in 5 mL GM and centrifuged at 200 x g for 3 min. The supernatant was discarded, and the cells were washed with 5 mL DPBS. After another centrifugation step and removal of the supernatant, the cells were carefully resuspended in 5 ml BSC-GM and counted with Invitrogen Countess Automated Cell Counter.

For short-term analysis SCs (800 cells/well = approx. 2000 cells/cm^2^) were plated in 96-well tissue culture plates (Greiner #655180) containing 100 µl BSC-GM with 1.5 µg recombinant human Vitronectin (FisherScientific #15134499)/cm^2^. After 24 h of incubation (day 1) at 37 °C and 5% CO_2_, the cells were rinsed with DPBS (ThermoFisher #14190250), BSC-GM was removed, screening medium (B8 or B9) and screening components were added (see the list of components and their sourcing in Supplementary Table 1). On indicated days Presto Blue assay was performed. On day 3 after Presto Blue assay screening components were added again into the fresh medium. Over the weekend cells were covered with 200 µl medium, instead of 100 µl. On the last day, the cells were rinsed with DPBS and frozen at -80 °C.

Long-term analysis was performed in 6-well plates (Greiner #657160) containing 1 mL BSC-GM with 1.5 µg recombinant human Vitronectin (FisherScientific #15134499)/cm^2^. After 24 h of incubation (day 1) at 37 °C and 5% CO_2_, the cells were rinsed with DPBS (ThermoFisher #14190250), BSC-GM was removed and screening medium with or without screening components/stabilizers were added. At 60–70% confluency cell were counted using Invitrogen Cell Countess and the total viable cells of three replicates were further analysed. Cells were then passaged using Trypsin-EDTA (0.25%) (ThermoFisher #25200056) and plated in either growth medium or B8 along with 1.5 µg recombinant human Vitronectin /cm^2^. After 24 h stabilizers and screening components were added, and cells were analyzed for proliferation and differentiation.

### Cultivation of CHO-K1 cell line

The CHO-DUXB11 EpoFc cell line was previously described by Taschwer et al.^64^. Cells were kept in suspension culture in an orbital shaking CO_2_ incubator at 7% CO_2_, 37 °C and 220 rpm in TPP^®^ TubeSpin bioreactor tubes (Merck, USA). Cells were passaged to a seeding density of 0,15 − 0,2 × 10^6^ cells/mL in 10 mL working volume every 3–4 days. To determine cell concentration and viability, a 500 µL sample of suspension culture was measured using trypan blue staining with the Vi-CELL™ XR cell viability analyzer (Beckman Coulter, USA).

For the CHO-DUXB11 EpoFC cell line CD-CHO medium (Gibco™, Fisher Scientific, USA) supplemented with 0.2% Anti-clumping agent (Gibco™, Fisher Scientific, USA), was used as the standard GM.

### Presto blue assay

To measure the metabolic activity, which correlates with the number of cells, Presto Blue assay was performed throughout exponential growth phase (days 1–6) and higher confluency (days 7–10). Experiments were performed in 96-well plate format according to the instruction manual. Shortly, 10 µL Presto Blue reagent (ThermoFisher #A13262) was added to the remaining 90 µl of medium in each well and incubated at 37 °C for 1 h. After incubation, the medium was transferred to a fresh 96-well plate (Greiner #655101) and the fluorescence at 560 nm (excitation) and 590 nm (emission) was measured with a plate reader (BioTek SynergyMx). Depending on the time point of the assay, cells were covered with 100 or 200 µL of fresh B8 or B9 medium and further incubated.

### Hoechst assay

In order to support Presto Blue assay data and to assess if the proliferation induction led to higher cell density under contact inhibition conditions at higher confluency (days 7–10), we used Hoechst 33,258 assay to assess the quantity of DNA per well, which directly correlates with the cell number.

On the last day of every screening, the screening plates were frozen at -80 °C. After thawing to room temperature, 100 µL ddH_2_O were added per well and the plates were incubated at 37 °C for 1 h. The plates were then again frozen at -80 °C and thawed to room temperature. 100 µL of Hoechst dye reagent - consisting of 25 µL Hoechst 33258 stock solution (10 mg Hoechst 33258 (ThermoFisher #H1398)/mL in DMSO + ddH_2_O (1:4 v/v)) in 10 mL TNE buffer (10 mM Tris, 2 M NaCl, 1 mM EDTA, 2 mM sodium azide, pH 7.4), were added. After 15 min of incubation at room temperature, the fluorescence was measured at 352 nm (excitation) and 461 nm (emission).

### Viscosity measurements

Viscosity of the buffers and media with/without stabilizers was measured using Anton Paar Rheometer MCR 502 following instruction manual and calculated as a function of [shear stress, Pa]/[shear rate, 1/s] over 100 measuring points.

### NanoTemper thermophoresis assay

Monolith His-Tag Labeling RED-tris-NTA 2nd Generation kit (Cat#MO-L018) was used to label His-tagged rhHGF via binding to its His-tag according to the instruction manual. Thermophoresis assay was performed on MonolithX (MM-235), with Monolith Premium Capillary at 25 °C with 25 nM His-tagged rhHGF with MC in a range of concentrations from 0.25 g/L to 0.00032 mg/L.

### Statistical analysis

For statistical analysis of Presto Blue and Hoechst assays, the data was tested for significance using ordinary one-way ANOVA followed by Dunnett’s multiple comparisons test, performed using GraphPad Prism version 10.2.3. Statistical significance was reached when *p* < 0.05 (*), *p* < 0.01 (**), *p* < 0.001 (***) and *p* < 0.0001 (****). Non-significant results (*p* ≥ 0.05) were marked as “n.s.”. The error bars in the graphs represent the standard deviation.

#### DoE

The design space is spanned by the 6 stabilizers given in Supplementary Table 2. Trials of the experiment were only able to be run at the grid points within the design space specified by the 8 stabilizer concentrations (see Supplementary Tables 2 and 3). A space-filling design^36^ with 48 trials was created and each trial was shifted to the nearest grid point afterwards. The resulting concentrations of each of the 6 stabilizers were summed up and the 8 trials with the largest sums were excluded from the design to avoid possible adverse effects of higher viscosity.

### Linear and linear mixed effects models

For each day (4, 6, and 8) separately we fitted linear second order regression models with the 6 stabilizers as explanatory variables. The largest models could contain all 6 stabilizers, their quadratic terms and their two-way interactions as regressors. The best model was chosen by a stepwise forward regression using Akaike’s information criterion (AIC) to decide whether an additional regressor enters the model or to stop the estimation.

To analyze the measurements of days 4–8 together in a single model we fitted a second order linear mixed effects model to our data additionally including the day and its quadratic term as regressors. Again, the best model was chosen by a stepwise forward regression using AIC.

## Electronic supplementary material

Below is the link to the electronic supplementary material.

Supplementary Material 1

## Data Availability

The data supporting the results reported in the article is available upon reasonable request from Aleksandra Fuchs aleksandrafuchs@acib.at or Harald Pichler harald.pichler@acib.at.
