# Supplementary material for: Low-cost food-grade alternatives for serum albumins in FBS-free cell culture media
Source: Sci Rep. 2025 May 1;15:15296. doi: 10.1038/s41598-025-99603-7 (PMC12045953; doi:10.1038/s41598-025-99603-7)
Supplement: Supplementary file 1 — Supplementary Material 1 [file 41598_2025_99603_MOESM1_ESM.docx]

**Title**

Low-cost food-grade alternatives for serum albumins in FBS-free cell culture media

**Authors**

Lisa Schenzle^1#^, Kristina Egger^1#^, Bernhard Spangl^2^, Mohamed Hussein^1,6^, Atefeh Ebrahimian^1,3^, Harald Kuehnel^3^, Frederico C. Ferreira^4,5^, Diana M. C. Marques^4,5^, Beate Berchtold^1^, Nicole Borth^6^, Aleksandra Fuchs^1^*, Harald Pichler^1,7^

*^1^acib - Austrian Centre of Industrial Biotechnology, Graz, Austria.*

*^2^Institute of Statistics, BOKU University, Vienna, Austria.*

*^3^**Department of Applied Life Science, Bioengineering, FH-Campus Wien, Vienna, Austria.*

*^4^Department of Bioengineering and Institute for Bioengineering and Biosciences, Instituto Superior Técnico, Universidade de Lisboa, Av. Rovisco Pais, 1049-001 Lisbon, Portugal.*

*^5^Associate Laboratory i4HB—Institute for Health and Bioeconomy, Instituto Superior Técnico, Universidade de Lisboa, Av. Rovisco Pais, 1049-001 Lisbon, Portugal.*

*^6^Department of Biotechnology, BOKU University, Vienna, Austria.*

*^7^Institute of Molecular Biotechnology, Graz University of Technology, NAWI Graz, BioTechMed Graz, Austria.*

**^#^Co-first authors**

These authors contributed equally.

***Correspondence**

Aleksandra Fuchs

[aleksandrafuchs@acib.at](mailto:aleksandrafuchs@acib.at)

Petersgasse 14/V,

Graz 8010, Austria

**
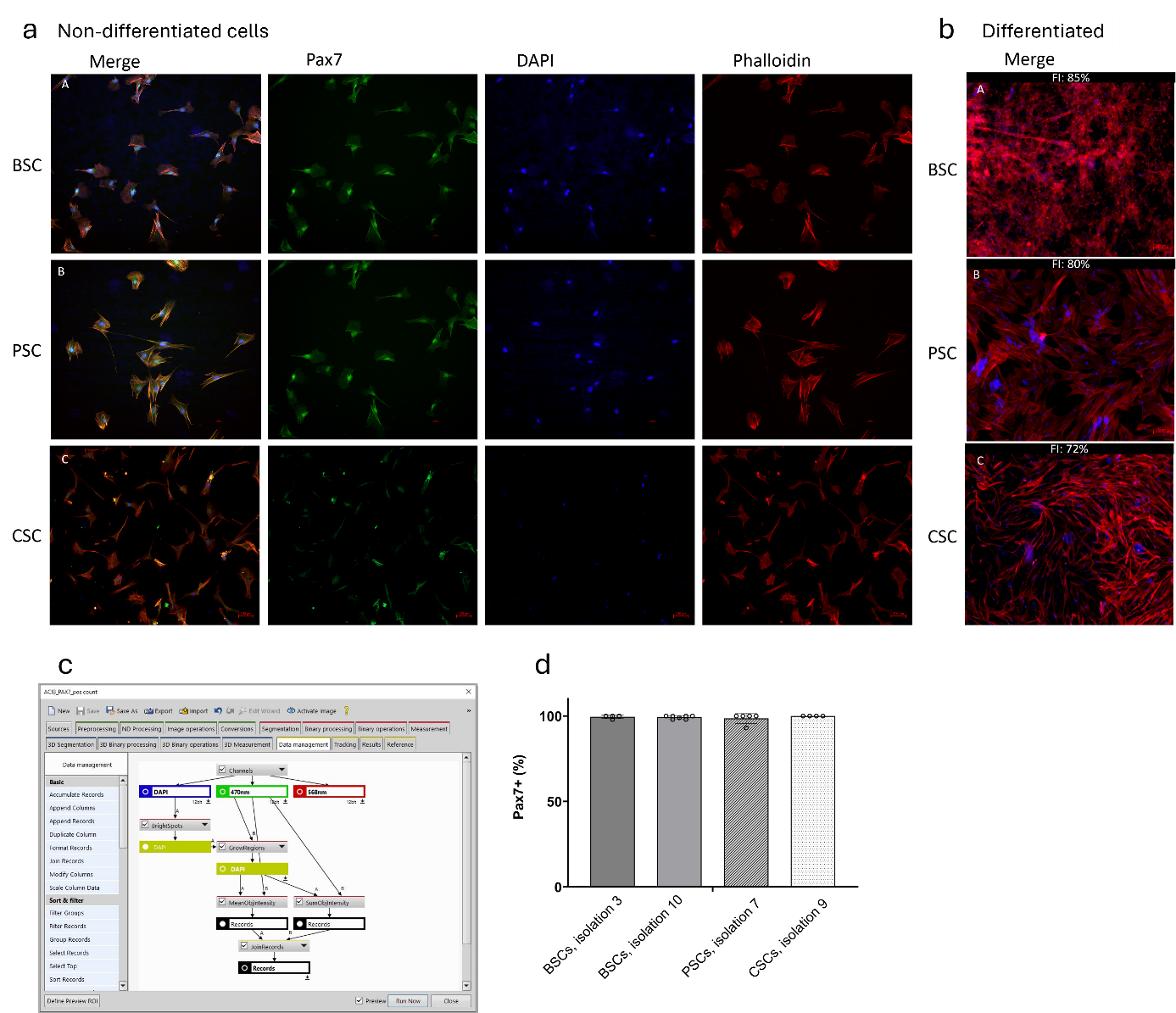
**

**Supplementary Figure 1:** Differentiated and non-differentiated cells of different species after isolation. BSC = Bovine Satellite Cells; PSC = Porcine Satellite Cells; CSC = Chicken Satellite Cells. Cells were isolated from sacrificed animals using a standard protocol described by Stout et al.^1^ Satellite cell (SCs) were propagated for several days after isolation, and **(a)** stained for the typical marker of non-differentiated satellite cells Pax7 labelled with Alexa Fluor™ 488 (green) and actin (red); or **(b)** differentiated as described in the methods section and stained for the typical marker of differentiated satellite cells - actin using phalloidin labelled with Alexa Fluor™ 594 (red). Nuclei were visualized using DAPI (blue). FI – fusion index calculated as a percentage of nuclei within the multinucleated myotubes, containing at least 2 nuclei. A range of 250-1700 nuclei were counted for each sample. The DAPI count was performed by the Zeiss Zen tool for cell counting. Using **(c)** the following decision tree of the Nikon NIS-software, **(d)** Pax7+ cell quantification of the isolations used in the study was perfromed. n= 4-7 images per isolation with 20-50 nuclei/image. Scale bar = 100 µm. Lens: EC Plan-Neofluar 20x; Exposure DAPI: 20 ms, Phalloidin: 300 ms. Pax7: 200 ms, FI = Fusion Index.

**Supplementary Table 1:** Cost comparison and sourcing of different GFs and stabilizing agents used in this study.

| **Compound** | **Article Nr** | **Cost** | **Costs €/L medium** |
| --- | --- | --- | --- |
| rhHGF | ab245957 | 225 €/ 25 µg | 45 €/L |
| rhPDGF-BB | Genscript #Z02529 | 2165 €/ 1 mg | 11 €/L |
| Estrogen (17β-Estradiol) | Sigma E2758-250MG | 28.40 €/ 250 mg | 0.000568 €/L |
| rm Wnt3a | 1324-WN-002 | 270 €/ 2 µg | 670 €/L |
| rh Wnt-5b | R&D 7347-WN-025/CF | 469 €/ 25 µg | 93.8 €/L |
| rh IL-6 | Peprotech 200-06 | 2600 €/ 1 mg | 13 €/L |
| rhAlbumin | Oryzogen OsrHSA | € 25.000- /kg | 0.8 g/L → 20.- €/L |
| DL-Alanine, ≥99%, FCC, FG | Sigma W381810-10KG-K | € 61.2 /kg | 0.4495 g/L (5 mM) → 0.0275 €/L |
| Methyl cellulose viscosity: 4,000 cP | Sigma M0512-1KG | € 328.- /kg | 0.1125 g/L → 0.037 €/L |
| Starch (corn) | Merck # S4126-5KG (5 kg) | € 42.8 /kg | 0.4 g/L → 0.017 €/L |
| Starch (rice) | Merck # S7260-1KG (1 kg) | € 76.50 /kg | - |
| D-Sorbitol | Roth # 6213.7 (25 kg) | € 13.56 /kg | 0.4 g/L → 0.005 €/L |
| D-Mannitol | Roth # 4175.2 (5 kg) | € 83.8 /kg | 0.1 g/L → 0.008 €/L |
| Inulin | Thermo # 457105000 | € 1,142.- /kg | - |
| Locust bean gum | Merck # G0753-1KG | € 163.- /kg | - |
| Cyanoflan | Gift from Dr. Rita Mota^2,3^ | Costs unknown | 0.8 g/L → Costs unknown |

**
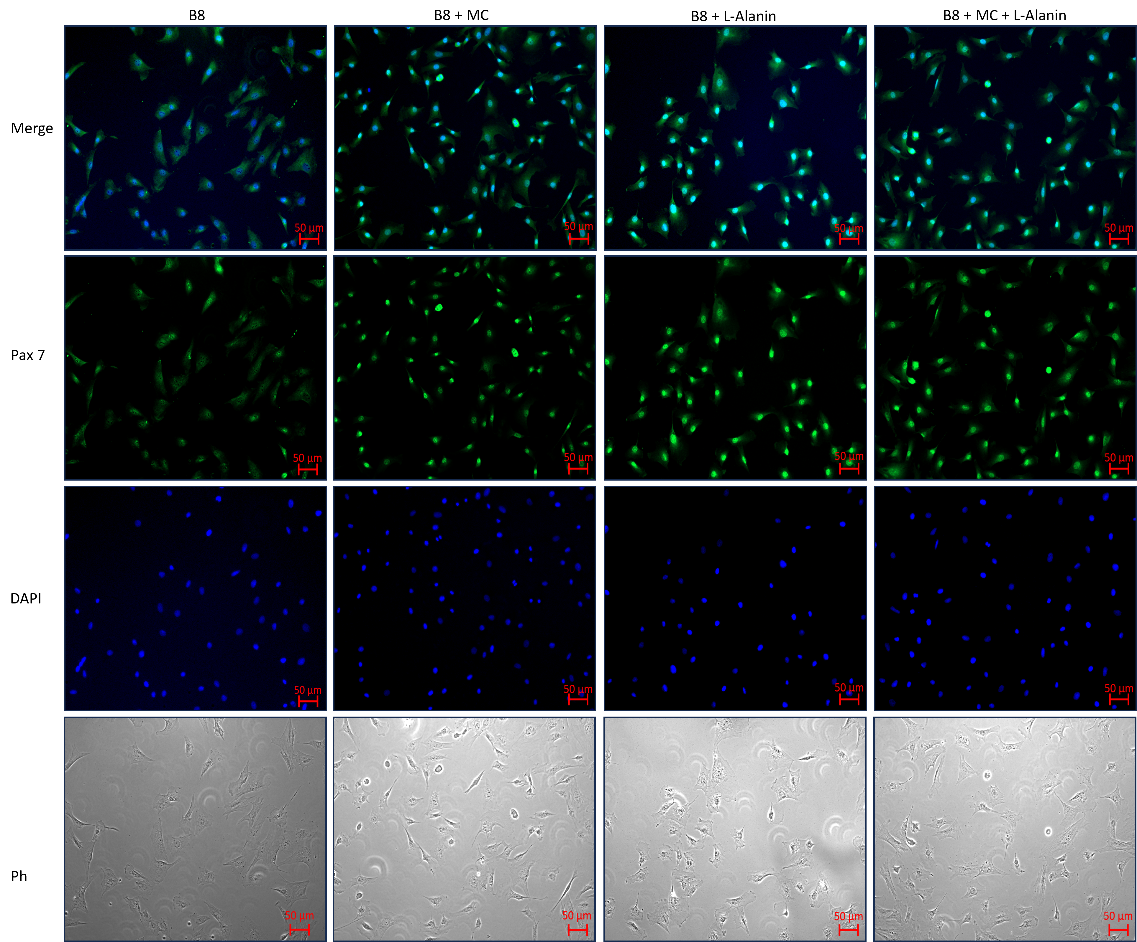

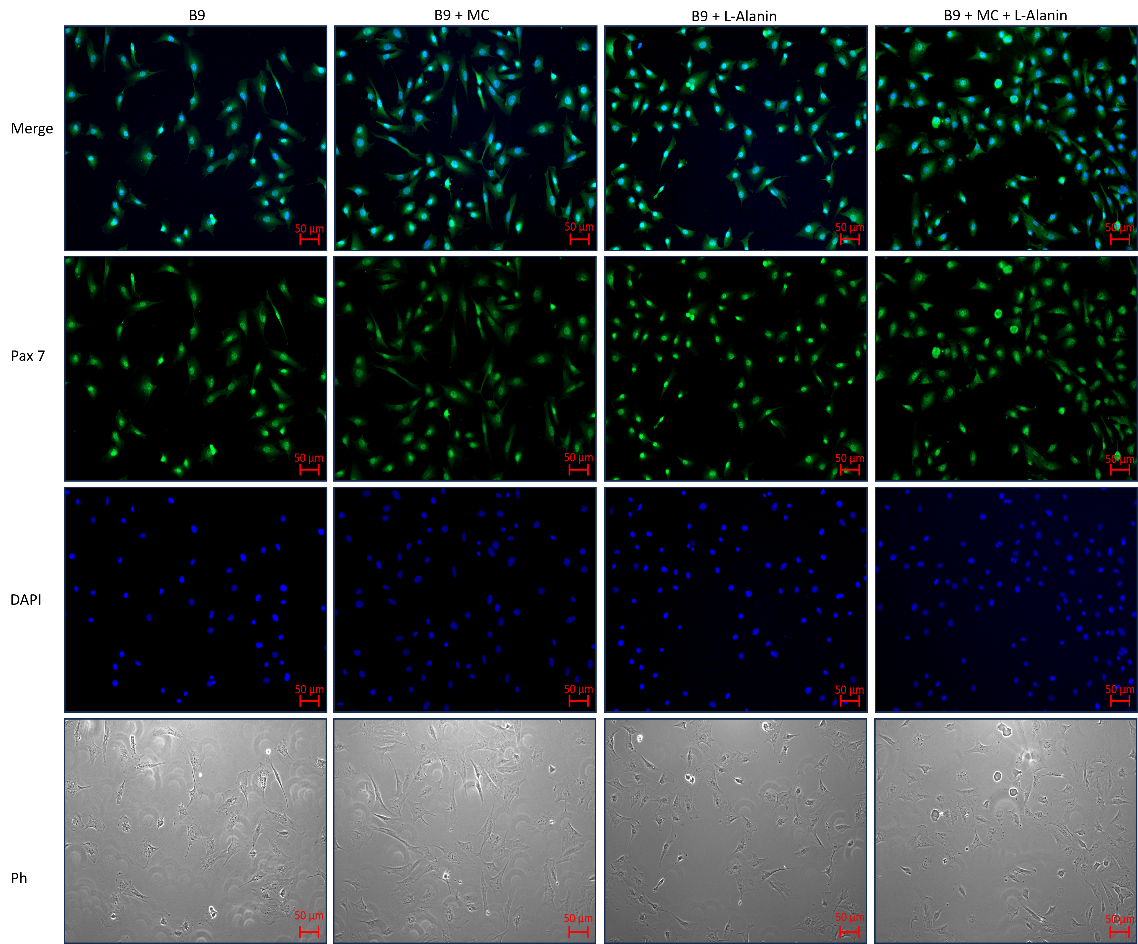
**

**Supplementary Figure 2. Microscopy images of non-differentiated BSCs do not reveal morphological changes upon addition of stabilizers.** 2000 BSCs/cm^2^ were seeded on day 0 in BSC-GM, and changed on day 1 to either B8 or B9 medium, with methyl cellulose (MC) and/or alanine (ALA) added to indicated end-concentrations. On day 3 cells were fixed and stained for the typical marker of non-differentiated satellite cells Pax7 labelled with Alexa Fluor™ 488 (green). Nuclei were visualized using DAPI (blue). Scale bar = 50 μm. Microscope Zeis Axio Imager. Lens: EC Plan-Neofluar 20x; Exposure DAPI: 20 ms, Pax7: 300 ms, GM = growth medium.

| \| 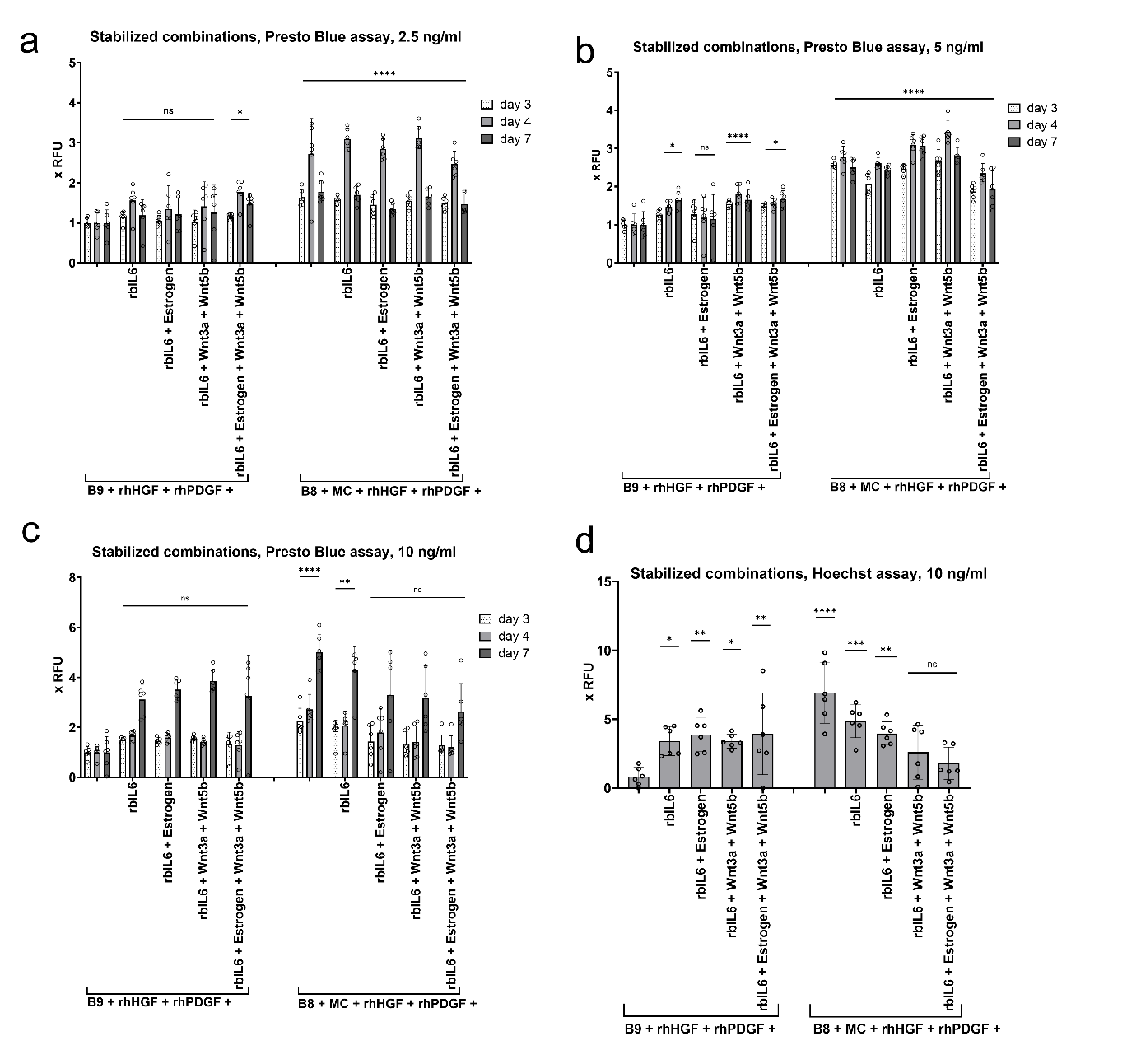 \| \| --- \|   **Supplementary Figure 3: Comparison of stabilization effects of HSA and MC for best proliferation inducing combinations in B8/B9 medium**. 2000 BSCs/cm^2^ were seeded on day 0 in BSC-GM, and changed on day 1 to B8 medium, stabilized with either 0.8 g/L human serum albumin (HSA) (B9) or 0.1125 g/L methyl cellulose (MC). Best proliferation inducing combinations were added on day 1 and day 3 to an end concentration of 2.5, 5 or 10 ng/ml, whereas stabilizers were added each time with medium exchange. Presto Blue assay was performed on days 3, 4, and 7 **(a-c)**, and Hoechst assay on day 7 **(d)**. Obtained values were normalized to B9 + rhHGF + rhPDGF. n=6 and repeated at least twice; statistical significance was calculated by one-way ANOVA combined with Dunnett test for day 4 (Presto Blue) or for day 7 (Hoechst), comparing all samples with B9 + rhHGF + rhPDGF, and is indicated by asterisks, which are p < 0.05 (*), p <0.01 (**), p < 0.001 (***), p < 0.0001 (****). |
| --- | --- |

| 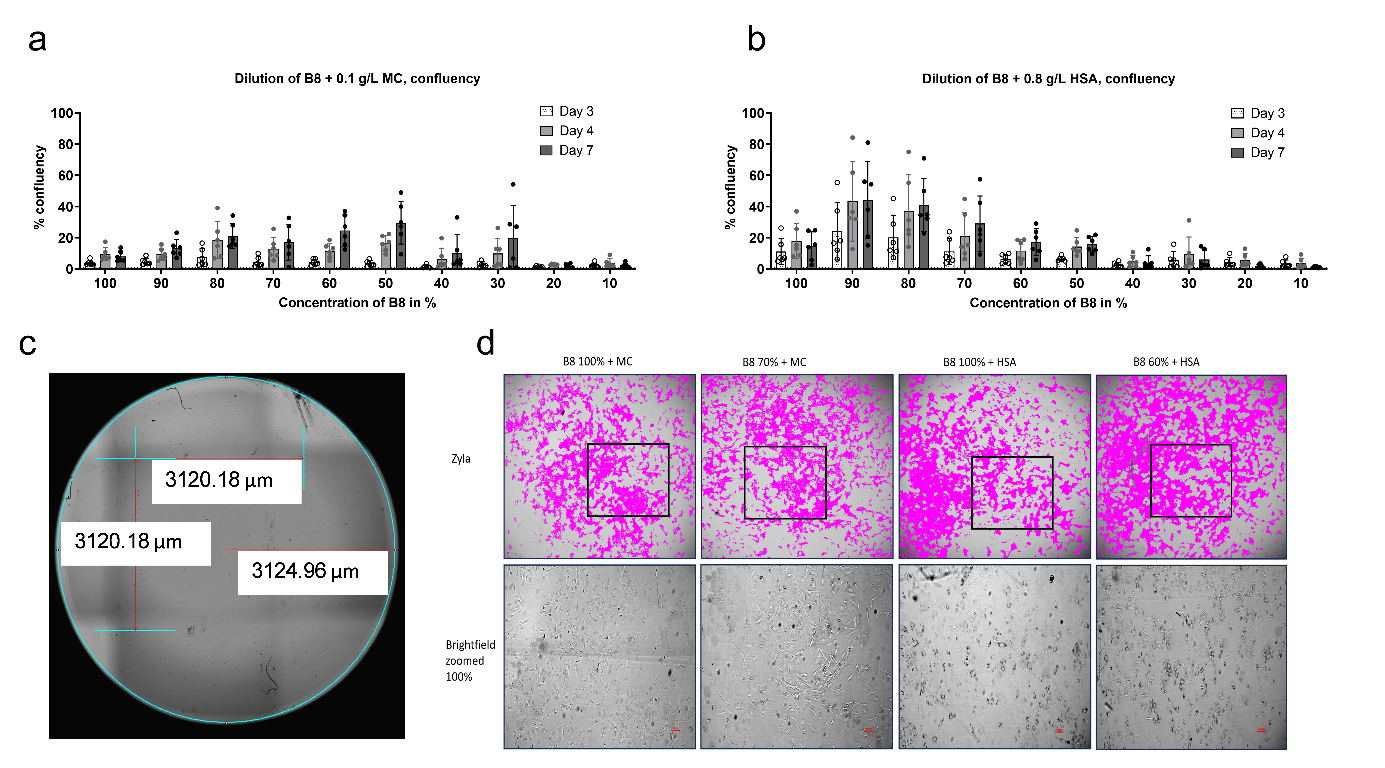 |
| --- |

**Supplementary Figure 4:** **Cell confluency upon non-specific stabilization of cultured medium components.** 2000 BSCs/cm^2^ were seeded on day 0 in BSC-GM, and changed on day 1 to either B8 + 0.1 g/L MC or B9 medium. Confluency was calculated based on images taken on indicated days **(a-b).** The central well area of 3120.18 µm x 3120.18 µm (approx. 1/3 of the well), as shown in **(c),** was photographed to avoid lens effect of the medium at the wells’ edges. Microscope Nikon Eclipse Ti2 was used with objective Nikon Plan Apo 4x; NA:0,20. NIS-Elements JOBS module with General Analysis was used to identify the area occupied by the cells, and its relation to the whole recorded area was calculated. It is presented in percent **(a)** for B8 + 0.1 g/L MC and **(b)** for B9. n=6. **(d)** Representative images of the conditions studied in **(a-b)** are shown. Zyla - area occupied by cells detected by image analysis, black rectangle – area represented below as “Brightfield zoomed 100%”, showing no morphological changes upon medium dilution in both B8 + 0.1 g/L MC and B9 medium.

| 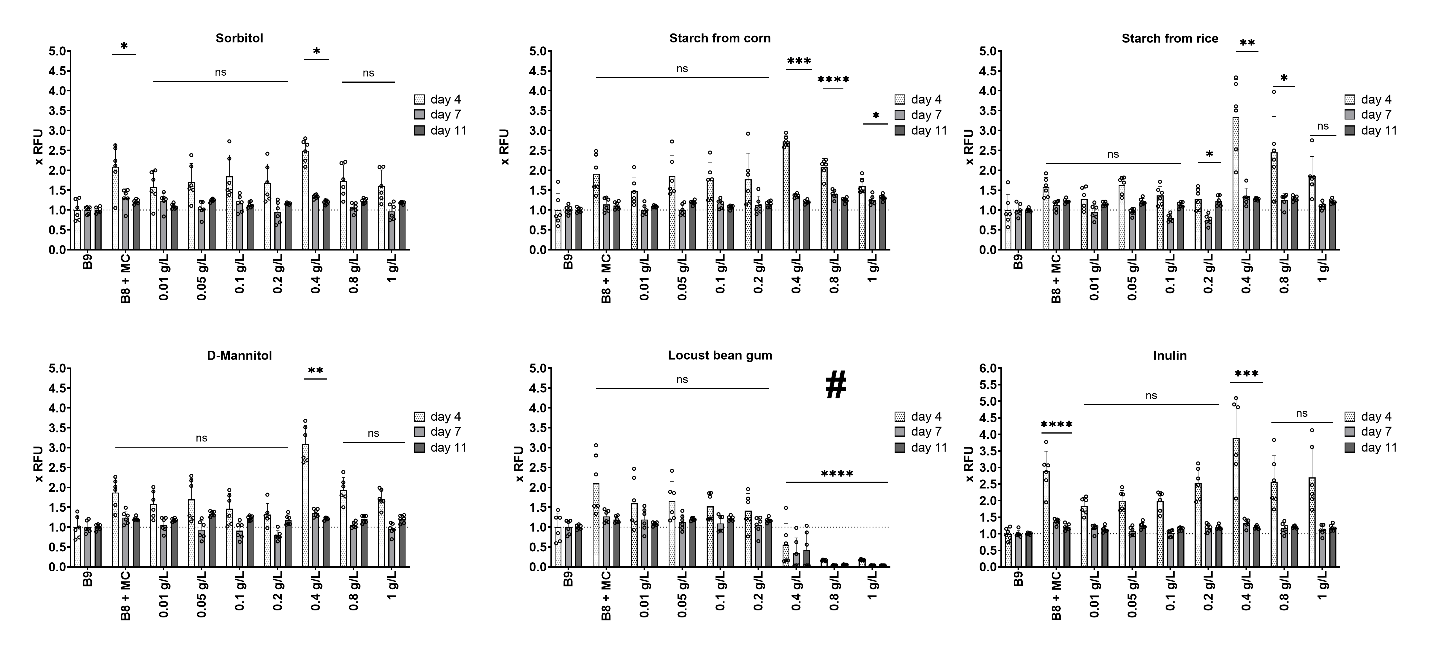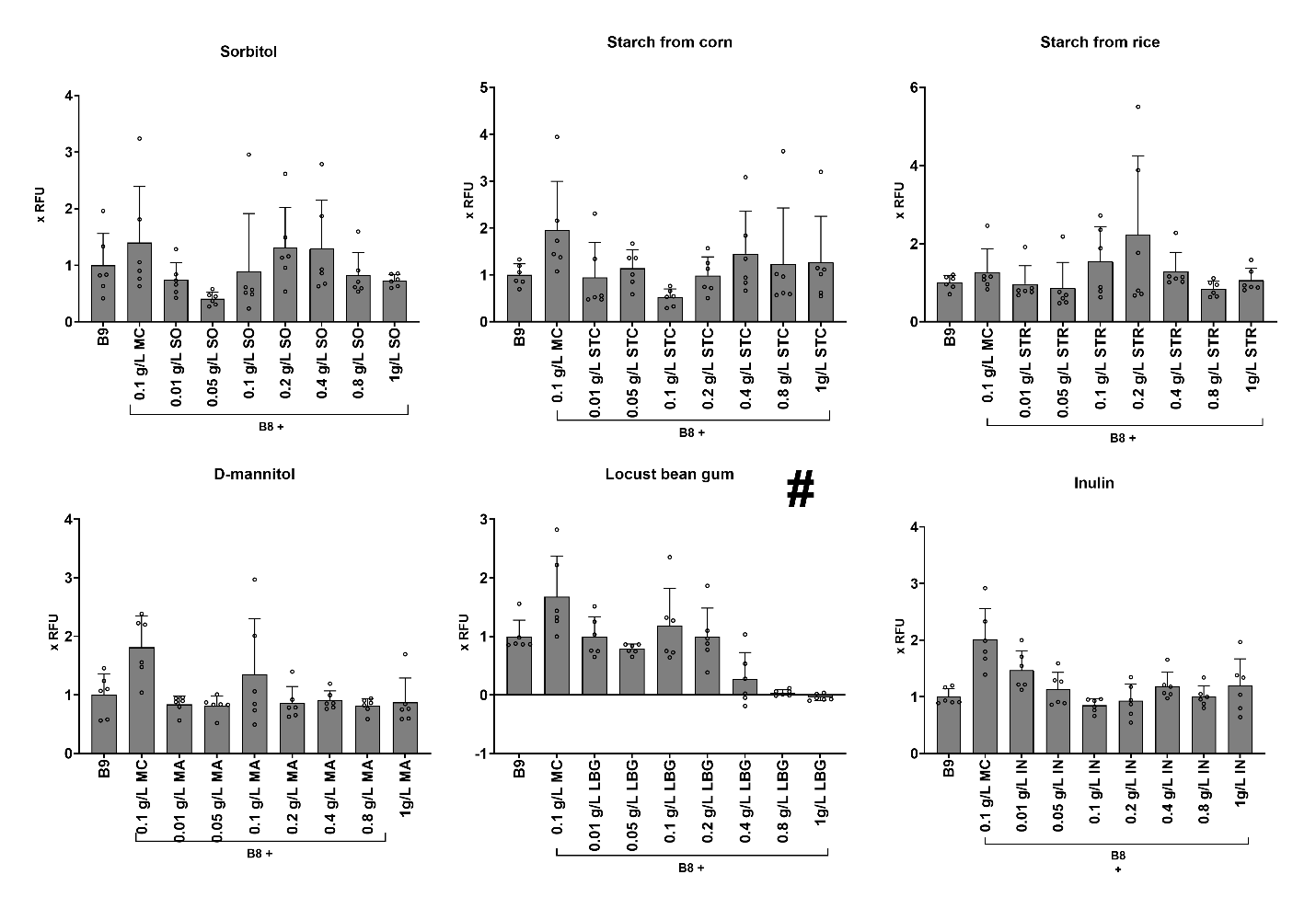 |
| --- |
|  |

**Supplementary Figure 5:** **Non-specific stabilization of cultured medium components by new potential stabilizers.** 2000 BSCs/cm^2^ were seeded on day 0 in BSC-GM, and changed on day 1 to the designated medium. B8 with 0.1 g/L methyl cellulose (MC) and B9 medium were used as controls. Stabilizers were added to indicated end-concentrations with every medium exchange. Presto Blue assay was performed on indicated days, and Hoechst assay was performed on the last day of the experiment. Obtained values were normalized to BSCs in B9. **#**LBG concentrations were probably not accurate because of aggregation during storage. n=6 and repeated at least twice; statistical significance was calculated by one-way ANOVA combined with Dunnett test for day 7, comparing all samples to 100% B9, and is indicated by asterisks, which are p < 0.05 (*), p <0.01 (**), p < 0.001 (***), p < 0.0001 (****).

**Supplementary Table 2:** Concentration ranges of stabilizers in DoE setup.

| Starch from corn (STC), HSA, g/L | Methyl cellulose (MC),  g/L | Sorbitol (SO), Mannitol (MA), Inulin (IN), g/L |
| --- | --- | --- |
| 0 | 0 | 0 |
| 0.005 | 0.002 | 0.01 |
| 0.01 | 0.01 | 0.05 |
| 0.05 | 0.02 | 0.1 |
| 0.1 | 0.04 | 0.2 |
| 0.2 | 0.08 | 0.4 |
| 0.4 | 0.16 | 0.8 |
| 0.8 | 0.2 | 1 |

**Supplementary Table 3:** DoE experimental setup for short term proliferation experiments with concentrations of stabilizers in g/L. Proliferation experiments were performed with BSCs as described in methods section, with Presto Blue as a reporting assay on days 4, 6 and 8. Starch from corn (STC), human serum albumin (HSA), methyl cellulose (MC), sorbitol (SO), mannitol (MA), inulin (IN). Each combination was performed in 6 replicates.

| 96-well plate # | Combination # | Stabilizer concentration, g/L | | | | | |
| --- | --- | --- | --- | --- | --- | --- | --- |
|  |  | SO | IN | MA | STC | HSA | MC |
| 1 | 1 | 0.01 | 0.01 | 0.01 | 0.005 | 0.005 | 0.002 |
|  | 2 | 0.05 | 0.05 | 0.05 | 0.01 | 0.01 | 0.01 |
|  | 3 | 0.1 | 0.1 | 0.1 | 0.05 | 0.05 | 0.02 |
|  | 4 | 0.2 | 0.2 | 0.2 | 0.1 | 0.1 | 0.04 |
|  | 5 | 0.8 | 0 | 0.05 | 0.1 | 0.8 | 0.002 |
|  | 6 | 0 | 0.8 | 0.2 | 0.005 | 0.8 | 0.08 |
|  | 7 | 0.2 | 0.4 | 1 | 0 | 0.005 | 0.01 |
|  | 8 | 1 | 0 | 0.01 | 0.01 | 0.05 | 0.04 |
| 2 | 9 | 0.01 | 0.8 | 0.05 | 0.8 | 0.1 | 0 |
|  | 10 | 1 | 0.01 | 0.1 | 0.4 | 0 | 0.01 |
|  | 11 | 0.05 | 0.2 | 1 | 0.005 | 0.05 | 0.16 |
|  | 12 | 1 | 0.05 | 0.8 | 0.005 | 0.2 | 0 |
|  | 13 | 0.2 | 1 | 0.01 | 0.05 | 0.4 | 0 |
|  | 14 | 0.8 | 0.01 | 0.4 | 0 | 0.05 | 0.2 |
|  | 15 | 0.4 | 0 | 0.1 | 0.8 | 0.01 | 0.16 |
|  | 16 | 0 | 0.4 | 0.01 | 0.4 | 0.01 | 0.2 |
| 3 | 17 | 0.1 | 1 | 0.05 | 0.4 | 0.005 | 0.08 |
|  | 18 | 0 | 0.1 | 1 | 0.01 | 0.4 | 0.002 |
|  | 19 | 0.8 | 0.05 | 1 | 0.1 | 0 | 0.08 |
|  | 20 | 0.4 | 1 | 0 | 0.005 | 0.01 | 0.02 |
|  | 21 | 0.2 | 0.1 | 0.05 | 0 | 0.8 | 0.16 |
|  | 22 | 0.1 | 0.05 | 0 | 0.8 | 0.4 | 0.08 |
|  | 23 | 0.1 | 0 | 0.8 | 0.1 | 0.005 | 0.2 |
|  | 24 | 0.01 | 0.8 | 0 | 0.05 | 0.8 | 0.01 |
| 4 | 25 | 0.2 | 0.01 | 1 | 0.2 | 0.05 | 0 |
|  | 26 | 0.8 | 0.8 | 0.2 | 0.05 | 0.01 | 0 |
|  | 27 | 0.2 | 0 | 0.4 | 0.005 | 0.4 | 0.01 |
|  | 28 | 0.1 | 0.2 | 0.4 | 0.8 | 0 | 0.002 |
|  | 29 | 0.05 | 0.8 | 0.01 | 0.2 | 0 | 0.02 |
|  | 30 | 0 | 0.05 | 0.2 | 0.8 | 0.005 | 0.02 |
|  | 31 | 0.05 | 1 | 0.2 | 0 | 0.2 | 0.002 |
|  | 32 | 0.4 | 0.2 | 0.1 | 0.01 | 0 | 0.2 |
| 5 | 33 | 0.01 | 0.05 | 0.1 | 0.1 | 0.2 | 0.2 |
|  | 34 | 0.1 | 0.8 | 0 | 0.01 | 0.1 | 0.2 |
|  | 35 | 0.01 |  | 0.8 | 0 | 0.01 | 0.04 |
|  | 36 | 0.4 | 0.1 | 0 | 0.4 | 0.1 | 0.002 |
|  | 37 | 1 | 0.2 | 0 | 0.2 | 0.005 | 0.16 |
|  | 38 | 0.05 | 0.1 | 0.2 | 0.2 | 0.8 | 0 |
|  | 39 | 0.01 | 1 | 0.4 | 0.05 | 0 | 0.16 |
|  | 40 | 0 | 0.01 | 0.05 | 0.05 | 0.1 | 0.08 |

**Supplementary Table 4:** DoE analysis results of the normalized cell culture densities in short term proliferation experiments (see experimental setup in Supplementary Table 3). Starch from corn (STC), methyl cellulose (MC), human serum albumin (HSA), sorbitol (SO), mannitol (MA), inulin (IN).

| **Analysis of day 4 only (norm)** | | | | | | **Analysis of day 6 only (norm)** | | |  |  |  |
| --- | --- | --- | --- | --- | --- | --- | --- | --- | --- | --- | --- |
|  | **Estimate** | **Std. Error** | **t-value** | **p-value** |  |  | **Estimate** | **Std. Error** | **t-value** | **p-value** |  |
| Intercept | 2.7954 | 0.3082 | 9.071 | 1.33E-10 | *** | Intercept | 1.3976 | 0.1454 | 9.61 | 2.38E-11 | *** |
| MC | 12.0005 | 2.3943 | 5.012 | 1.66E-05 | *** | MC | 1.9192 | 0.8917 | 2.152 | 0.0383 | * |
| STC^2 | 6.1751 | 2.9517 | 2.092 | 0.044 | * | SO | -0.5411 | 0.2067 | -2.618 | 0.013 | * |
| HSA | -0.5522 | 0.7702 | -0.717 | 0.4783 |  | IN | -0.3863 | 0.1947 | -1.984 | 0.0552 | . |
| STC | -3.3032 | 2.3567 | -1.402 | 0.1701 |  | MA^2 | -0.3756 | 0.2012 | -1.866 | 0.0704 | . |
| MC:HSA | -15.9333 | 9.3608 | -1.702 | 0.0979 | . |  |  |  |  |  |  |
| --- |  |  |  |  |  | --- |  |  |  |  |  |
| Signif. codes: 0 ‘***’ 0.001 ‘**’ 0.01 ‘*’ 0.05 ‘.’ 0.1 ‘ ’ 1  Residual standard error: 0.9694 on 34 degrees of freedom  Multiple R-squared: 0.5693, Adjusted R-squared: 0.5059  F-statistic: 8.987 on 5 and 34 DF, p-value: 1.621e-05 | | | | | | Signif. codes: 0 ‘***’ 0.001 ‘**’ 0.01 ‘*’ 0.05 ‘.’ 0.1 ‘ ’ 1  Residual standard error: 0.4249 on 35 degrees of freedom  Multiple R-squared: 0.324, Adjusted R-squared: 0.2468  F-statistic: 4.194 on 4 and 35 DF, p-value: 0.007046 | | | | | |
| **Analysis of day 8 only (norm)** | | |  |  |  | **Analysis of day 4 to 8** | |  |  |  |  |
|  | **Estimate** | **Std. Error** | **t-value** | **p-value** |  |  | **Value** | **Std.Error** | **DF** | **t-value** | **p-value** |
| Intercept | 0.79335 | 0.06829 | 11.617 | 3.34E-13 | *** | Intercept | 12.619483 | 1.1501787 | 78 | 10.971759 | <0.0001 |
| MC | 0.66172 | 0.4896 | 1.352 | 0.18572 |  | dayExp | -3.178518 | 0.4049667 | 78 | -7.848838 | <0.0001 |
| SO | -0.33517 | 0.1133 | -2.958 | 0.00568 | ** | dayExp^2 | 0.207565 | 0.0335921 | 78 | 6.179001 | <0.0001 |
| IN | -0.78384 | 0.3965 | -1.977 | 0.05646 | . | MC | 4.988338 | 0.9152115 | 31 | 5.450476 | <0.0001 |
| MA^2 | -0.15828 | 0.08603 | -1.84 | 0.07481 | . | STC^2 | 1.88163 | 0.4085037 | 31 | 4.606152 | 0.0001 |
| IN^2 | 0.59827 | 0.40464 | 1.479 | 0.14875 |  | MA^2 | -0.313818 | 0.1949828 | 31 | -1.609463 | 0.1177 |
| MC:SO | 1.55959 | 1.16977 | 1.333 | 0.19158 |  | SO^2 | 0.3652 | 0.2520497 | 31 | 1.448923 | 0.1574 |
|  |  |  |  |  |  | MC:HSA | -8.262807 | 3.1337875 | 31 | -2.636684 | 0.013 |
| --- |  |  |  |  |  | STC:IN | -2.169967 | 0.6459205 | 31 | -3.359495 | 0.0021 |
| Signif. codes: 0 ‘***’ 0.001 ‘**’ 0.01 ‘*’ 0.05 ‘.’ 0.1 ‘ ’ 1  Residual standard error: 0.1795 on 33 degrees of freedom  Multiple R-squared: 0.4424, Adjusted R-squared: 0.3411  F-statistic: 4.364 on 6 and 33 DF, p-value: 0.002381 | | | | | | STC:SO | -3.233155 | 1.0025931 | 31 | -3.224792 | 0.003 |
|  |  |  |  |  |  | HSA:SO | -1.693671 | 0.6741099 | 31 | -2.512455 | 0.0174 |
|  |  |  |  |  |  |  |  |  |  |  |  |

**
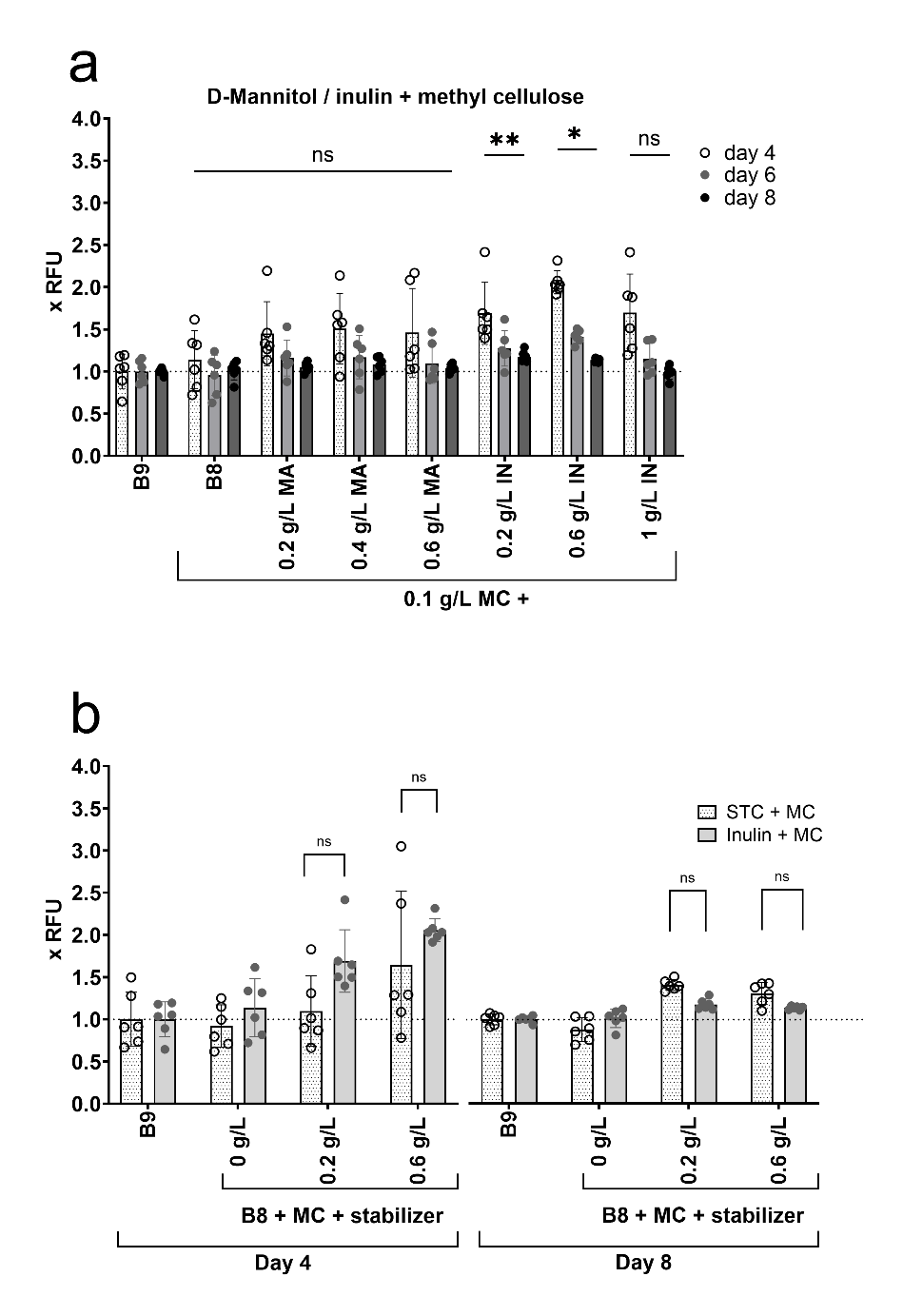
**

**Supplementary Figure 6:** **D-mannitol and Inulin as stabilizers in B8 + MC.** **(a-b)** 2000 BSCs/cm^2^ were seeded on day 0 in BSC-GM, and changed on day 1 to the designated medium. B8 with 0.1 g/L methyl cellulose (MC) and B9 medium were used as controls. Stabilizers were added to indicated end-concentrations with every medium exchange, MC always 0.1 g/L. Presto Blue assay was performed on indicated days. Obtained values were normalized to BSCs in B9. n=6 and repeated at least twice; statistical significance was calculated by one-way ANOVA combined with Dunnett test for day 8 **(a)** or day 4 and day 8 **(b)**, comparing all samples to 100% B9 **(a)**, and is indicated by asterisks, which are p < 0.05 (*), p <0.01 (**), p < 0.001 (***), p < 0.0001 (****). MA – D-mannitol, IN – inulin.


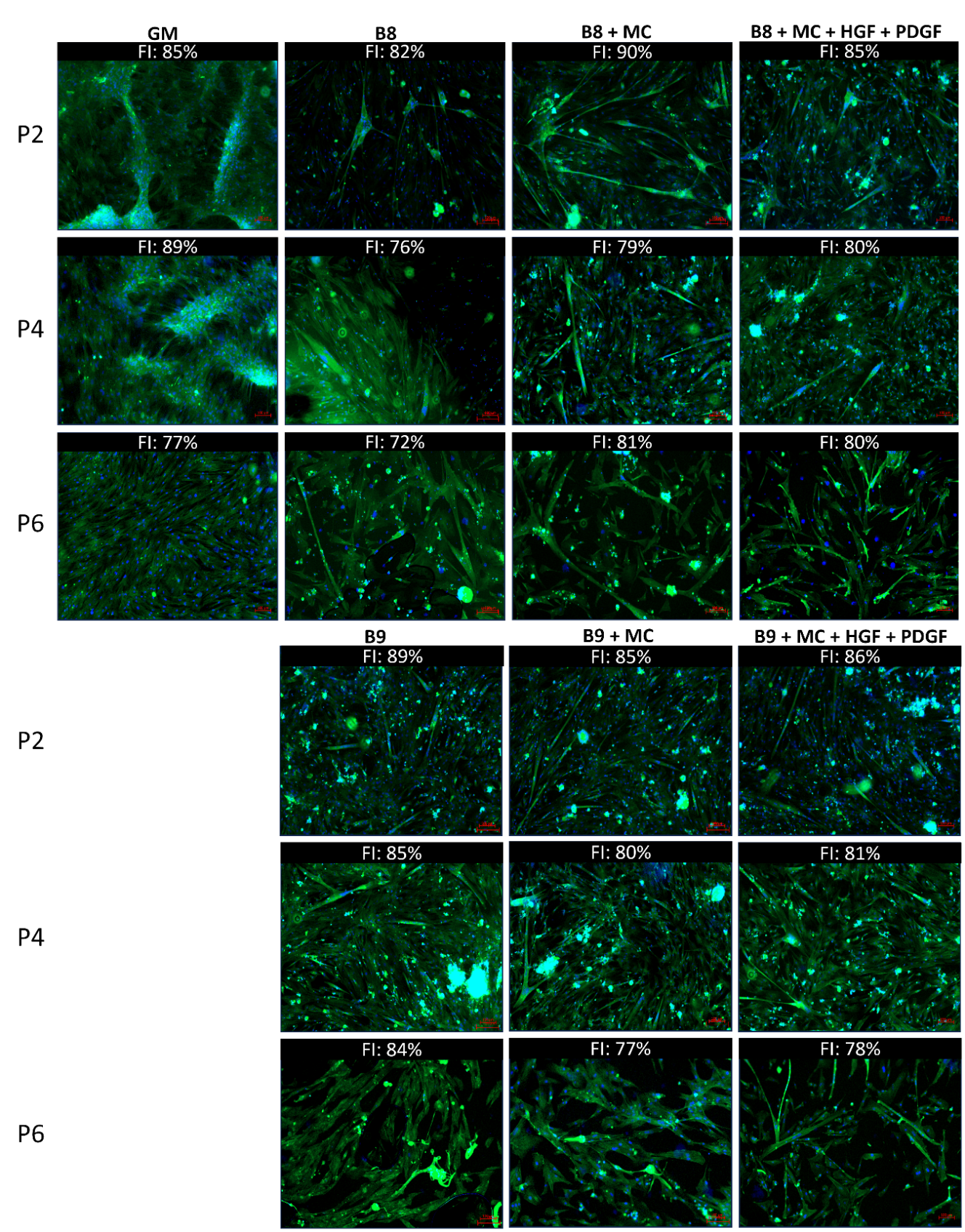


**Supplementary Figure 7: Desmin staining of long-term proliferation experiments with BSCs in stabilized B8 and B9.** Cells were cultured in 6-well plates in corresponding media, with 0.1 g/L MC and 2.5 ng/ml GFs rhHGF and PDGF added where indicated immediately upon splitting, whereas HSA was added 24h after the splitting. Cells were stained with trypan blue and counted upon passaging using Invitrogen Cell Countess, the number of viable cells and population doublings and doubling time are presented in **Figure 6**. Upon each passage, part of the cells was seeded for differentiation and IF staining (actin and desmin) and fusion indices were calculated based on the ratio of cells with at least two nuclei to all nuclei upon actin staining. A range of 250-1700 nuclei were counted for each sample. The count was performed by the Zeiss Zen tool for cell counting. Immunofluorescence staining was performed for nuclei (DAPI, blue), desmin and actin (phalloidin, presented in **Figure 6**). Cells were grown to confluency and differentiated for 10 days in a serum-free differentiation medium (see Materials and Methods), with addition of stabilizers, if they were present in propagation medium. Scale bar = 100 μm. Microscope Zeis Axio Imager. Lens: EC Plan-Neofluar 10x; Exposure DAPI: 20 ms, Phalloidin: 300 ms, Desmin: 300 ms. GM = growth medium, FI = Fusion Index.

| \| 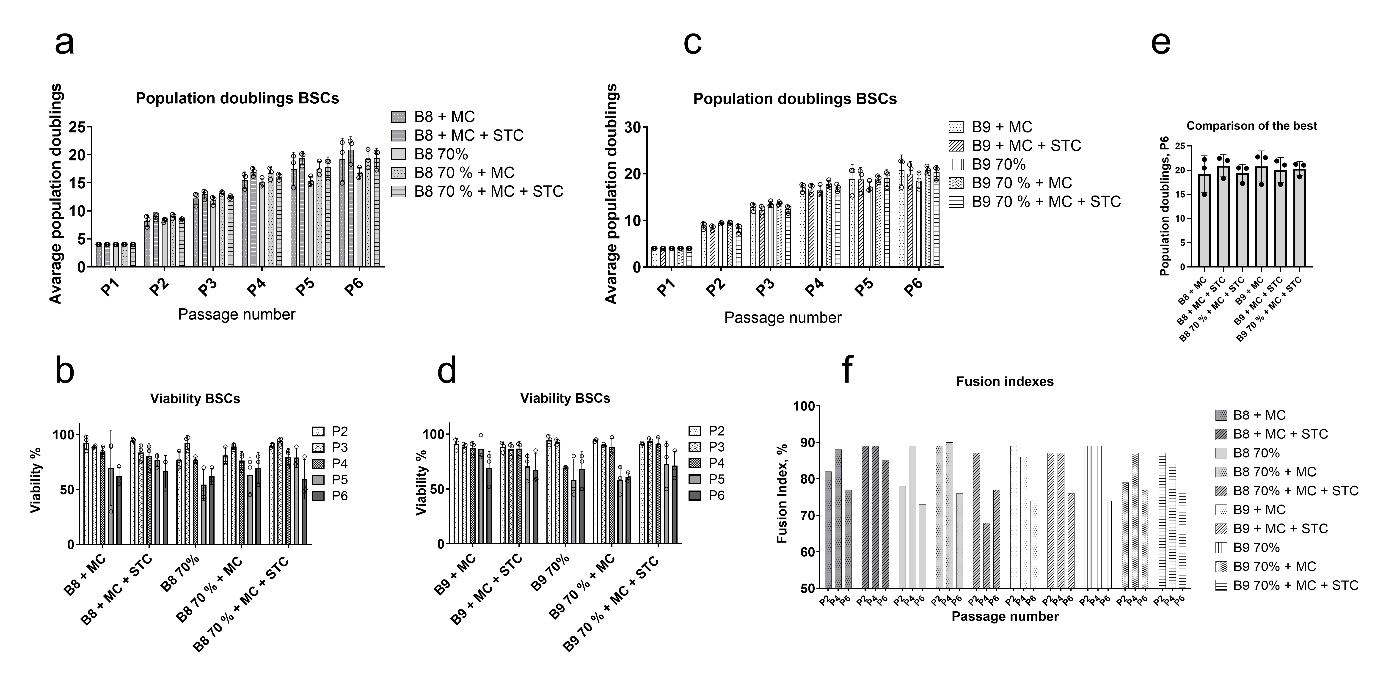 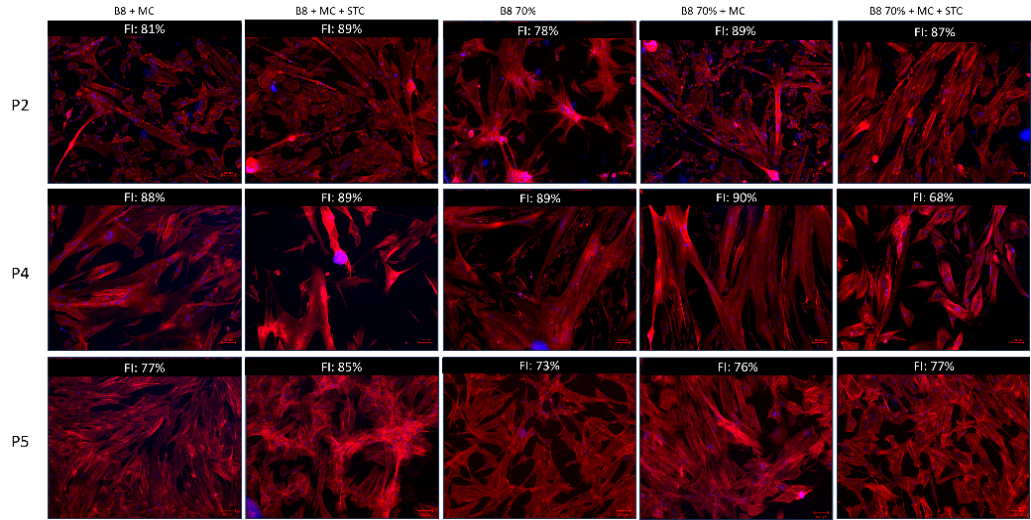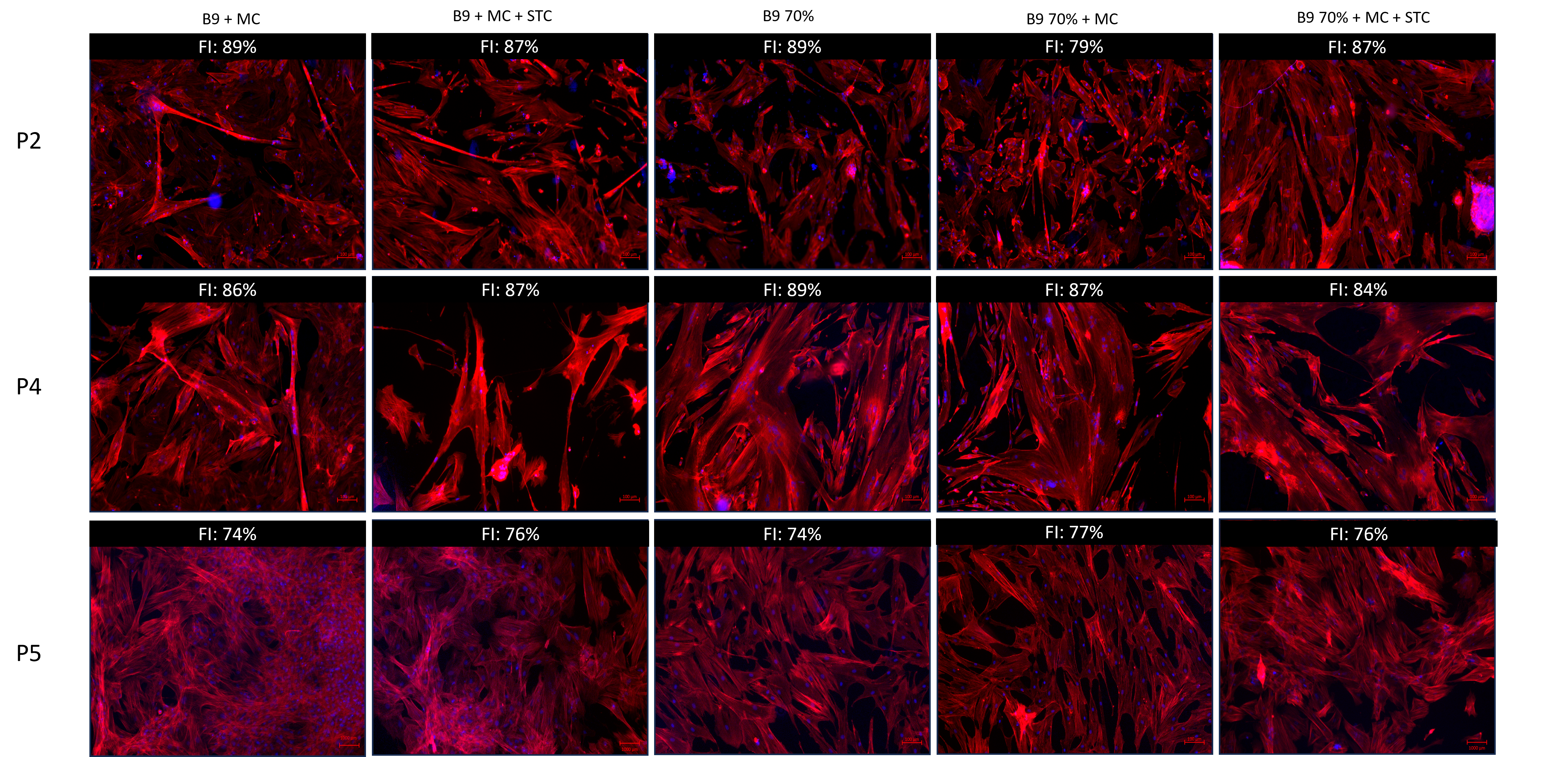 g \| \| --- \|  \|  \| \| --- \| \|  \| \| |
| --- | --- | --- | --- | --- |

**Supplementary Figure 8:** **Effect of STC and GF reduction on BSCs proliferation.** Cells were cultured in 6-well plates in corresponding media, with 0.1 g/L MC and 0.4 g/L STC added where indicated immediately upon splitting, whereas HSA was added 24 h after the splitting. Cells were stained with trypan blue and counted upon passaging using Invitrogen Cell Countess, and the number of viable cells **(b, d)** was used to calculate population doublings **(a, c)**. Average population doublings P1 = 4 was assessed based on cells count after isolation. Upon each passage, part of the cells was seeded for differentiation and IF staining **(****e)**, and fusion indexes were calculated based on the ratio of cells with at least two nuclei to all nuclei. A range of 250-1700 nuclei were counted for each sample. The count was performed by the Zeiss Zen tool for cell counting. **(f)** Immunofluorescence staining was performed for nuclei (DAPI, blue), desmin (not shown) and actin (phalloidin, red). Cells were grown to confluency and differentiated for 10 days in a serum-free differentiation medium (see Materials and Methods), with addition of stabilizers, if they were present in propagation medium. Scale bar = 100 μm. Microscope Zeis Axio Imager. Lens: EC Plan-Neofluar 10x; Exposure DAPI: 20 ms, Phalloidin: 300 ms. GM = growth medium, FI = Fusion Index.

| 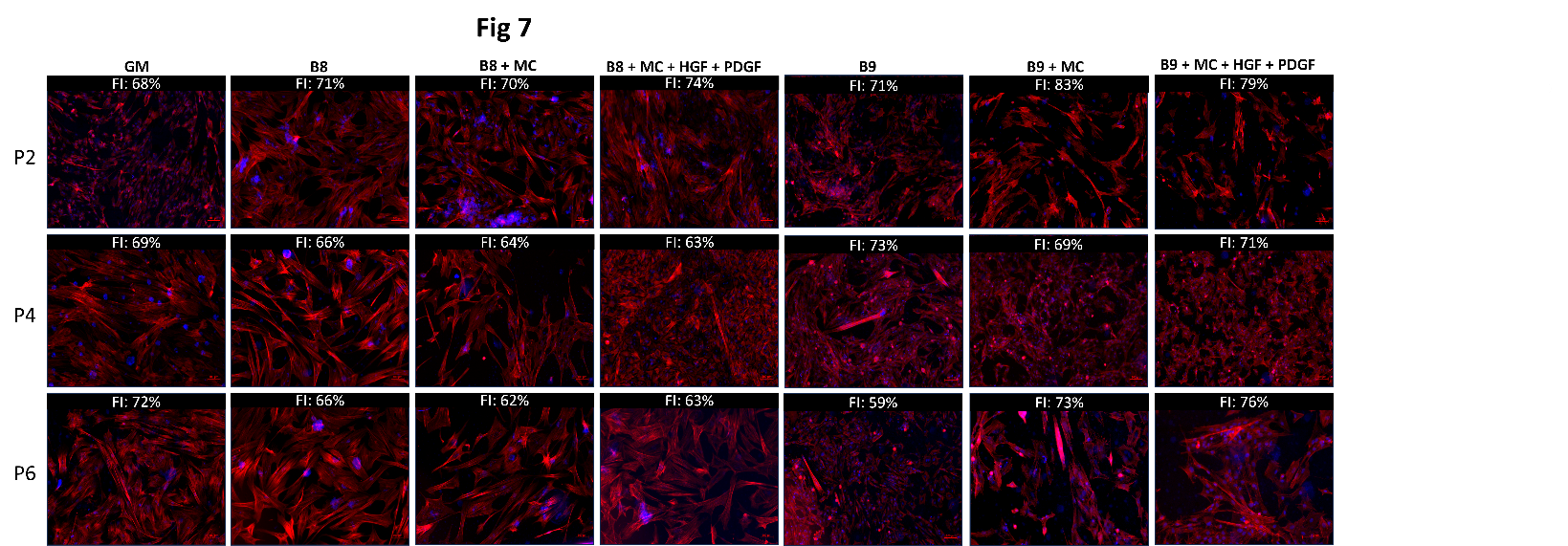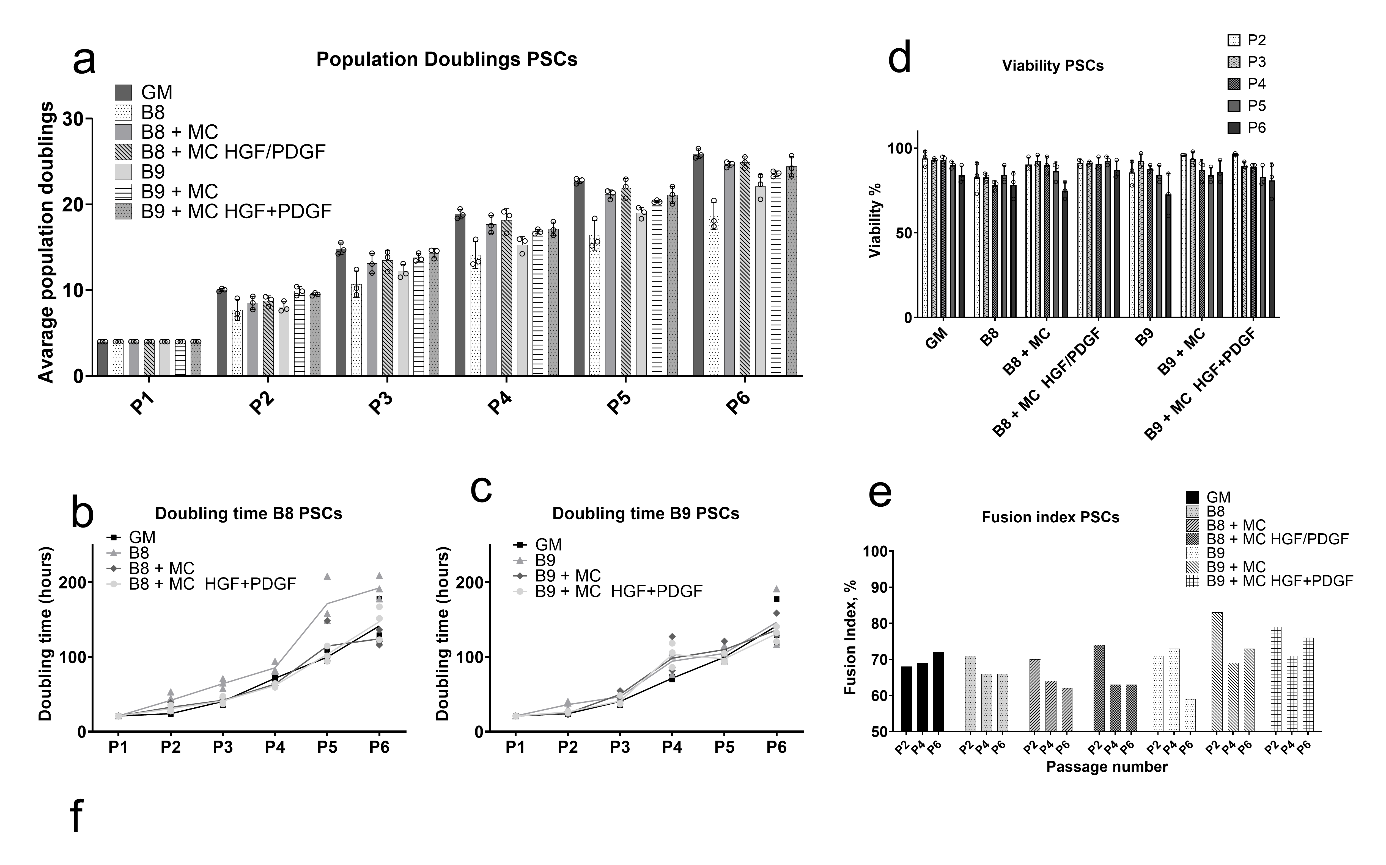 |
| --- |

**Supplementary Figure 9: Long term proliferation experiments with porcine satellite cells (PSCs) in stabilized B8 and B9.** Cells isolated from a 6 month old male Sus scrofa domestica were cultured in 6-well plates in corresponding media, with 0.1 g/L MC and 2.5 ng/ml GFs rhHGF and PDGF added where indicated immediately upon splitting, whereas HSA was added 24 h after the splitting. **(a)** Average population doublings P1 = 4 was assessed based on cells count after isolation. Cells were stained with trypan blue and counted upon passaging using Invitrogen Cell Countess, and the number of viable cells **(d)** was used to calculate population doublings and doubling time **(a-c)**. Upon each passage, part of the cells was seeded for differentiation and IF staining **(f)** and fusion indices **(e)** were calculated based on the ratio of cells with at least two nuclei to all nuclei. A range of 250-1700 nuclei were counted for each sample. The count was performed by the Zeiss Zen tool for cell counting. **(f)** Immunofluorescence staining was performed for nuclei (DAPI, blue), desmin (not shown) and actin (phalloidin, red). Cells were grown to confluency and differentiated for 10 days in a serum-free differentiation medium (see Materials and Methods), with addition of stabilizers, if they were present in propagation medium. Scale bar = 100 μm. Microscope Zeis Axio Imager. Lens: EC Plan-Neofluar 10x; Exposure DAPI: 20 ms, Phalloidin: 300 ms. GM = growth medium, FI = Fusion Index.

| \| 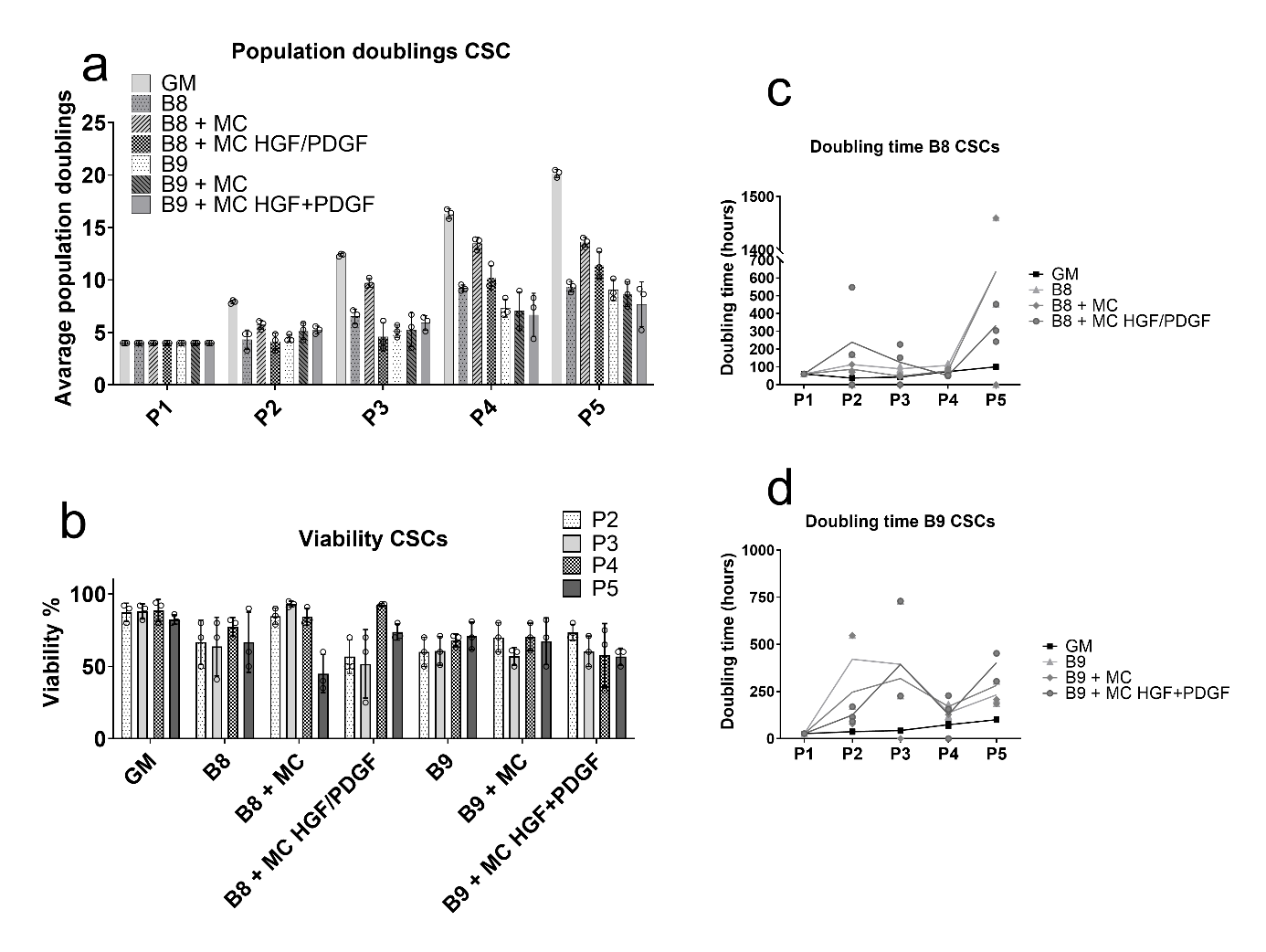 \| \| --- \| |
| --- | --- |

**Supplementary Figure 10: Long term proliferation experiments with chicken satellite cells (CSCs) in stabilized B8 and B9.** CSCs were cultured in 6-well plates in corresponding media, with 0.1 g/L MC and 2.5 ng/ml GFs rhHGF and PDGF added where indicated immediately upon splitting, whereas HSA was added 24 h after the splitting. **(a)** Average population doublings P1 = 4 was assessed based on cells count after isolation. Cells were stained using trypan blue and counted upon passaging using Invitrogen Cell Countess, and the number of viable cells **(b)** was used to calculate population doublings **(a)** and doubling time **(c-d)**.

|  |
| --- |


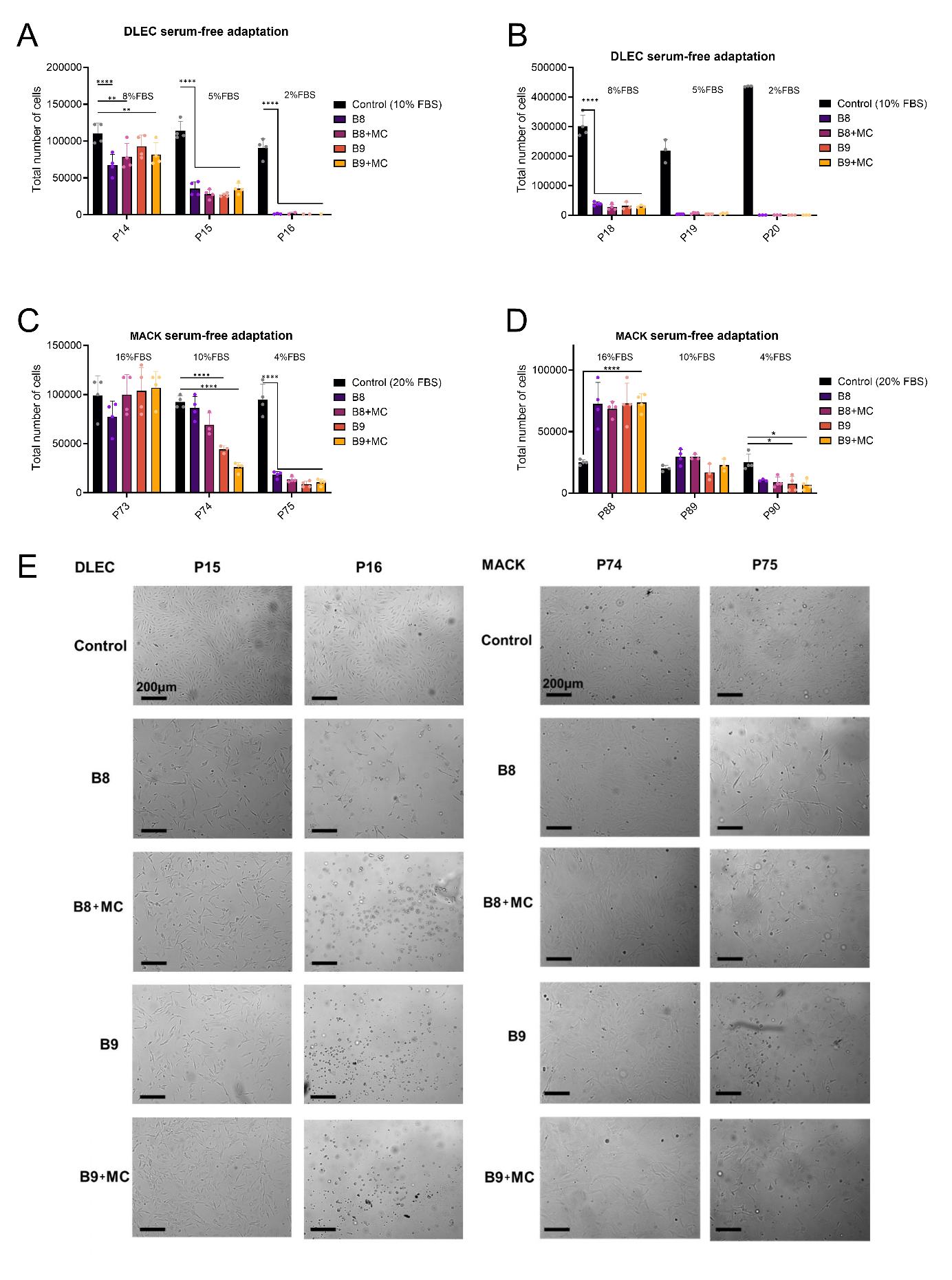


**Supplementary Figure 11:** Effect of stabilizers MC and HSA on propagation of DLEC and MACK1 cells. 10000 cells/cm^2^ were seeded on day 0 into designated medium. L15 + 10% FBS (DLEC) and L15 + 20% FBS (MACK1) were used as controls. Stabilizers were added to indicated end-concentrations with every medium exchange. Cell number was assessed using a haemocytometer and trypan-blue staining (N=2; n=8). Statistical significance was calculated by two-way ANOVA combined with Dunnett’s test, and is indicated by asterisks, which are p < 0.05 (*), p <0.01 (**), p < 0.001 (***), p < 0.0001 (****). Microscopical analysis of the cells was performed to compare the cells’ morphology throughout the experiment.

| 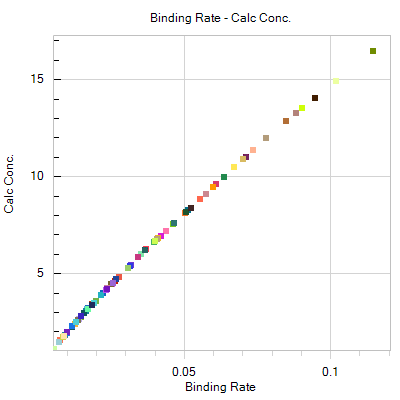 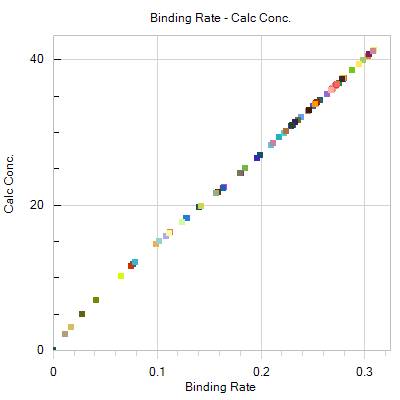 |
| --- |

**Supplementary Figure 12:** Binding rates of EPOFc in all tested samples was unaffected by the media supplementation with stabilizers, irrespective of the expression timepoint and stabilizer concentration. To assess the binding rate and concentration of the product, Octet RED96e (ForteBio) was used, sensor type - Protein A. **(a)** Samples in biological triplicates from 24 h - 96 h, **(b)** samples in biological triplicates from 96 h - 192 h expression in GM (CD CHO + 8 mM L-Gln + 0.2% ACA), GM + 0.1 g/L methyl cellulose and GM + 0.1g/L methyl cellulose + 0.8 g/L HSA.

| 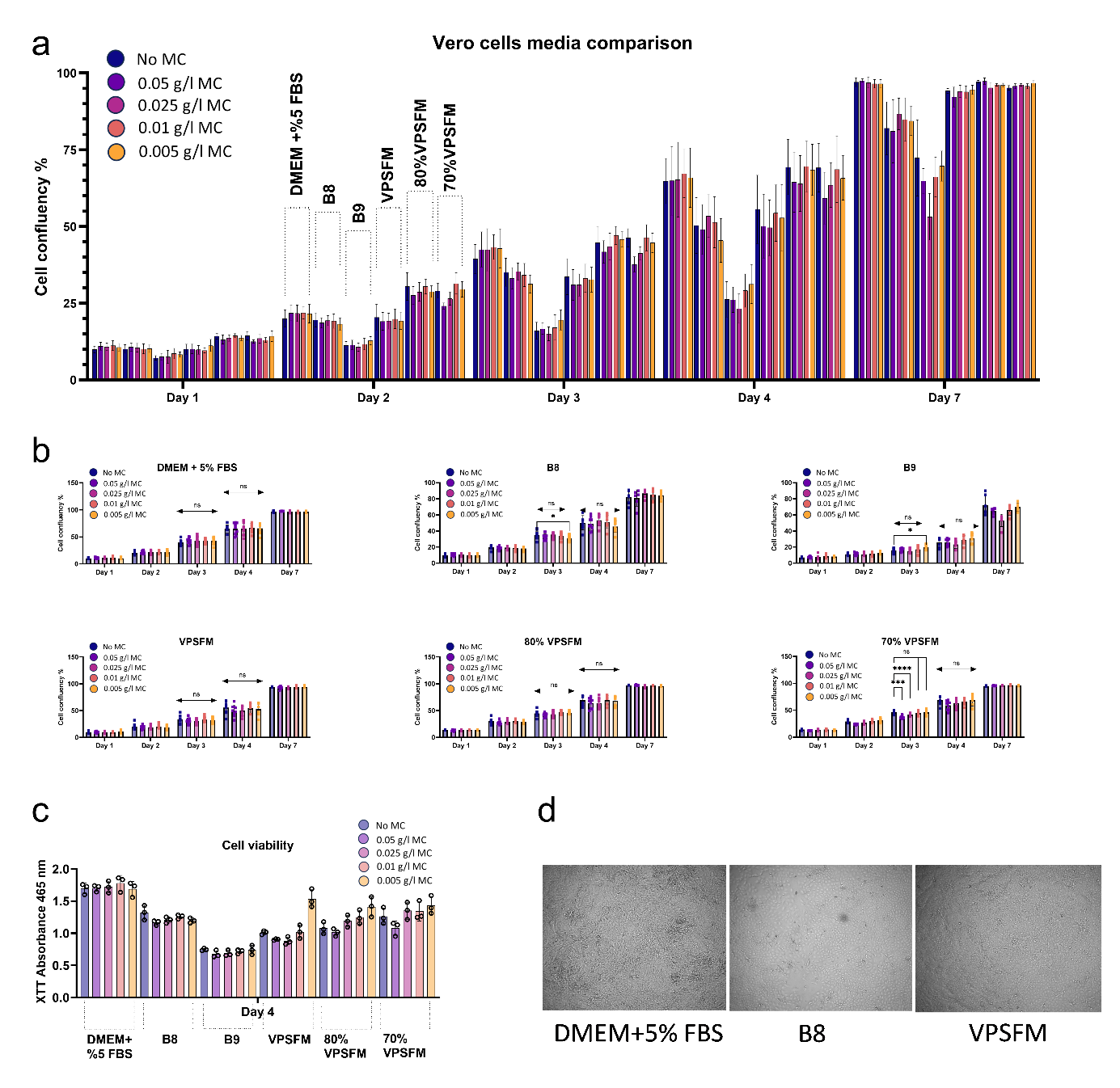 |
| --- |

**Supplementary Figure 13:** Effect of stabilizers MC and HSA on Vero cell proliferation. 10,000 cells/cm^2^ were seeded on day 0 into designated medium. DMEM + 5% FBS and commercially available medium VPSFM were used as controls. Stabilizers were added to the indicated final concentrations at the time of seeding and medium exchange with fresh medium (4^th^ day of culture). Confluence measurement was performed using live-cell imaging on TECAN device **(A-B)**, and the cell proliferation XTT assay (Roche) was performed according to the manufacturer instruction **(C)** in the designated days of the experiment. n=12; statistical significance was calculated by one-way ANOVA combined with the Kruskal-Wallis test for days 3 and 4, and is indicated by asterisks, which are p < 0.05 (*), p <0.01 (**), p < 0.001 (***), p < 0.0001 (****). Microscopical observation of the cells is performed to compare the cells morphology at 7^th^ day of culture **(D)**.

**Supplementary Table 5:** Assessment of medium price reduction potential for new medium stabilization agents on three serum-free media examples. The exchange of albumins (HSA/BSA) to MC+STC was assessed (light gray), as well as a potential exchange of Fetuin to MC+STC (dark gray). Basic medium costs – usually DMEM-F12 – were not considered. Bulk prices – where available – were used. Starch from corn (STC), human serum albumin (HSA), bovine serum albumin (BSA), methyl cellulose (MC).

| **Kolkmann *et al.***^4^ | | | | |
| --- | --- | --- | --- | --- |
| **Component** | **Component end concentration** | **Component bulk cost** | **Component cost**  **Euro/L medium** | **Link** |
| ITSE Animal-Free | 1% | 488.64 € per 100 mL | 4.89 | https://invitria.com/products/itse-af-recombinant-transferrin/ |
| Glutamax | 1% | 77.00 € per 100 mL | 7.7 | <https://www.thermofisher.com/order/catalog/product/35050061> |
| HSA | 5 mg/mL | 25 € per 1 g | 125 | <https://www.oryzogen.net/list/6.html> |
| Fibronectin | 10 µg/mL | 35 € per 1 mg | 350 | <https://www.oryzogen.net/list/6.html> |
| Hydrocortisone | 36 ng/mL | 305 € per 10g | 0.0011 | https://www.sigmaaldrich.com/ |
| Human IL-6 | 20 ng/mL | 2965 € per 1 mg | 59.3 | <https://www.peprotech.com/de/search?q=il+6> |
| Alpha linolenic acid | 1 µg/mL | 1680 € per 10 g | 0.168 | <https://www.sigmaaldrich.com/AT/de/product/sigma/l2376> |
| L-acsorbate-2-phosphat | 50 µg/mL | 513 € per 100 g | 0.26 | <https://www.sigmaaldrich.com/AT/de/product/sigma/49752> |
| FGF 2 | 10 ng/mL | 22 € per 1 mg | 0.22 | <https://www.oryzogen.net/list/6.html> |
| HGF | 5 ng/mL | 4,735.00 € per 1 mg | 23.68 | <https://www.peprotech.com/en/recombinant-human-hgf-insect-derived> |
| VEGF | 10 ng/mL | 150 € per 1 mg | 1.5 | <https://www.oryzogen.net/list/6.html> |
| IGF-1 | 100 ng/mL | 30 € per 1 mg | 3 | <https://www.oryzogen.net/list/6.html> |
| PDGF-BB | 10 ng/mL | 3,845.00 € per 1 mg | 38.45 | https://www.peprotech.com/en/recombinant-human-pdgf-bb |
|  |  | **Sum** | **614.16** |  |
|  |  | **Sum**  **HSA→MC+STC** | **489.21** | 0,4 g/L STC → 0.017 €/L <https://www.sigmaaldrich.com/AT/de/product/sial/s4126>  0.1125 g/L MC → 0.037 €/L <https://www.sigmaaldrich.com/AT/en/product/sigma/m0512> |
|  |  |  |  |  |
| **Stout *et al.*** ^1^ | | | | |
| Insulin | 20 µg/mL | 41,920 € per 100 g | 8.38 | <https://invitria.com/> |
| Ascorbic acid 2-phosphate | 200 µg/mL | 513 € per 100 g | 1.03 | <https://www.sigmaaldrich.com/AT/de/product/sigma/49752> |
| Transferrin | 20 µg/mL | 70 € per 1 g | 1.4 | https://www.oryzogen.net/list/6.html |
| Sodium selenite | 20 ng/mL | 240 € per 100 g | 0.000048 | https://www.sigmaaldrich.com/AT/de/product/sigma/s5261 |
| FGF2-G3 | 40 ng/mL | 22 € per 1 mg | 0.22 | https://www.oryzogen.net/list/6.html |
| TGFβ3 | 0.1 ng/mL | 1,235 € per 100 µg | 1.24 | [Recombinant human TGF beta 3 protein (Active) (ab269208) \| Abcam](https://www.abcam.com/en-at/products/proteins-peptides/recombinant-human-tgf-beta-3-protein-active-ab269208) |
| NRG1 | 0.1 ng/mL | 1,484.00 € per 1 mg | 0.15 | https://www.peprotech.com/en/recombinant-human-heregulin-1 |
| Sodium bicarbonate | 2438 µg/ml | 59.30 € per 500 g | 0.29 | <https://www.sigmaaldrich.com/AT/de/product/sigma/s5761> |
| HSA | 800mg/L | 25 € per 1 g | 20 | https://www.oryzogen.net/list/6.html |
|  |  | **Sum** | **32.70** |  |
|  |  | **Sum**  **HSA→MC+STC, % of total** | **12.75** | 0,4 g/L STC → 0.017 €/L <https://www.sigmaaldrich.com/AT/de/product/sial/s4126>  0.1125 g/L MC → 0.037 €/L <https://www.sigmaaldrich.com/AT/en/product/sigma/m0512> |
|  |  |  |  |  |
| **Skrivergaard *et al.***^5^ | | | | |
| FGF-2 | 2 ng/mL | 800 € per 1 mg | 32 | <https://www.peprotech.com/en/recombinant-human-fgf-basic-154-aa> |
| Fetuin | 600 µg/mL | 69.20 € per 100 mg | 415.2 | [https://www.sigmaaldrich.com/](https://www.sigmaaldrich.com/AT/de/search/f2379?focus=products&page=1&perpage=30&sort=relevance&term=f2379&type=product) |
| BSA | 75 µg/mL | 154 € per 100 mL 7.5% in DPBS (x100) | 15.4 | <https://www.sigmaaldrich.com/AT/de/product/sigma/a8412> |
| ITSE Animal-Free | 1% | 488.64 € per 100 mL | 4.89 | <https://www.thermofisher.com/order/catalog/product/51500056> |
| PDGF | 5 ng/ml | 3,845.00 € per 1 mg | 38.45 | https://www.peprotech.com/en/recombinant-human-pdgf-bb |
| HGF | 20 ng/ml | 4,735.00 € per 1 mg | 23.68 | <https://www.peprotech.com/en/recombinant-human-hgf-insect-derived> |
|  |  | **Sum** | **529.62** |  |
|  |  | **Sum**  **BSA→MC+STC** | **514.27** | 0,4 g/L STC → 0.017 €/L <https://www.sigmaaldrich.com/AT/de/product/sial/s4126>  0.1125 g/L MC → 0.037 €/L <https://www.sigmaaldrich.com/AT/en/product/sigma/m0512> |
|  |  | **Sum**  **BSA+Fetuin→MC+STC** | **99.07** |  |

**Supplementary References:**

1. Stout, A. J. *et al.* Simple and effective serum-free medium for sustained expansion of bovine satellite cells for cell cultured meat. *Commun. Biol.* **5**, 466 (2022).

2. Costa, R. *et al.* Biocompatibility of the Biopolymer Cyanoflan for Applications in Skin Wound Healing. *Mar. Drugs* **19**, 147 (2021).

3. Leite, J. P. *et al.* Cyanobacterium-Derived Extracellular Carbohydrate Polymer for the Controlled Delivery of Functional Proteins. *Macromol. Biosci.* **17**, (2017).

4. Kolkmann, A. M., Van Essen, A., Post, M. J. & Moutsatsou, P. Development of a Chemically Defined Medium for in vitro Expansion of Primary Bovine Satellite Cells. *Front. Bioeng. Biotechnol.* **10**, (2022).

5. Skrivergaard, S. *et al.* A simple and robust serum-free media for the proliferation of muscle cells. *Food Res. Int.* **172**, 113194 (2023).
